# Structure of Csx1-cOA_4_ complex reveals the basis of RNA decay in Type III-B CRISPR-Cas

**DOI:** 10.1038/s41467-019-12244-z

**Published:** 2019-09-20

**Authors:** Rafael Molina, Stefano Stella, Mingxia Feng, Nicholas Sofos, Vykintas Jauniskis, Irina Pozdnyakova, Blanca López-Méndez, Qunxin She, Guillermo Montoya

**Affiliations:** 1 Structural Molecular Biology Group, Novo Nordisk Foundation Centre for Protein Research, Faculty of Health and Medical Sciences University of Copenhagen, Blegdamsvej 3-B, Copenhagen, 2200 Denmark; 2 College of Life Science and Technology, Huazhong Agricultural University, 430070 Wuhan, China; 3 Archaea Center, Department of Biology, University of Copenhagen, Ole Maaløes Vej 5, Copenhagen Biocenter, DK-2200 Copenhagen N, Denmark; 4 The Novo Nordisk Foundation Center for Protein Research, Protein Structure & Function Programme, Faculty of Health and Medical Sciences, University of Copenhagen, Blegdamsvej 3B, 2200 Copenhagen, Denmark; 5 Microbial Technology Institute and State Key Laboratory of Microbial Technology, Shandong University, 72 Binhai Road, Jimo, 266237 Qingdao, Shandong P. R. China

**Keywords:** X-ray crystallography, CRISPR-Cas systems, Cryoelectron microscopy

## Abstract

Type III CRISPR-Cas multisubunit complexes cleave ssRNA and ssDNA. These activities promote the generation of cyclic oligoadenylate (cOA), which activates associated CRISPR-Cas RNases from the Csm/Csx families, triggering a massive RNA decay to provide immunity from genetic invaders. Here we present the structure of *Sulfolobus islandicus* (Sis) Csx1-cOA_4_ complex revealing the allosteric activation of its RNase activity. SisCsx1 is a hexamer built by a trimer of dimers. Each dimer forms a cOA_4_ binding site and a ssRNA catalytic pocket. cOA_4_ undergoes a conformational change upon binding in the second messenger binding site activating ssRNA degradation in the catalytic pockets. Activation is transmitted in an allosteric manner through an intermediate HTH domain, which joins the cOA_4_ and catalytic sites. The RNase functions in a sequential cooperative fashion, hydrolyzing phosphodiester bonds in 5′-C-C-3′. The degradation of cOA_4_ by Ring nucleases deactivates SisCsx1, suggesting that this enzyme could be employed in biotechnological applications.

## Abstract (web summary)

Type III CRISPR-Cas RNases from the Csm and Csx families are activated by cyclic oligoadenylates (cOA). Here the authors present the cOA bound *Sulfolobus islandicus* Csx1 structure, which forms a hexamer and reveal an allosteric mechanism for the activation of Csx1 RNase.

## Introduction

CRISPR-Cas systems provide adaptive immunity to bacteria and most archaea against mobile genetic elements such as invading plasmids and viruses, via an interference mechanism^1–4^. They rely on CRISPR arrays, which are short repetitive DNA segments interspersed with unique spacer sequences generated during encounters with invading nucleic acids; hence, constituting a genetic record of previous infections. Type III systems incorporate the *cas10* gene^5^, which codes for a multidomain protein^6^. This system is further divided into at least four subtypes: III-A, III-B, III-C, and III-D. Among them, several III-A and III-B systems have been characterized. Subtype III-A modules contain  four additional proteins (Csm2–Csm5) and target plasmid DNA in vivo^3^. Subtype III-B modules contain five or six additional proteins, Cmr1 and Cmr3-Cmr7, and four of them (Cmr1, Cmr3, Cmr4, and Cmr6) are RNA-binding proteins. These systems cleave RNA in vitro^7,8^, and in contrast with all other characterized CRISPR-Cas systems, the Csm and Cmr systems are unique among CRISPR-Cas effectors in that they mediate both RNA and DNA interference^9,10^, the latter of which is transcription-dependent^11^. In addition, *csm6/csx1*-like gene family encode poorly characterized RNases frequently associated with Type III CRISPR-Cas systems^5,12^. The Csm6/Csx1 RNases are thought to non-specifically degrade foreign transcripts and play auxiliary or sometimes essential functions during Type III interference even though they are not part of the effector complex^11,13–15^. Csm6 genes are preferentially associated to type III-A, while Csx1 is found in types III-B, III-C, and III-D with no clear link to a particular subtype^5,16^. Recently, it has been shown that the Cas10 subunit of the Csm and Cmr complexes synthesizes cyclic oligoadenylates (cOAs), which act as second messengers to allosterically regulate the Cas-associated ribonucleases Csm6^17,18^ and Csx1^19,20^, triggering robust interference in the presence of an invader. Although the structures of Csx1 from *P. furiosus* (Pfu)^21^ and *S. solfataricus* (Sso) (PDB:2I71), and Csm6 from *T. thermophilus* (Tt)^14^ and *T. onnurineus (To)*^22^ have been determined, the heterogeneity of the abundant Type III CRISPR-Cas systems^5,16^, the presence of Ring nucleases^23^ and divergences in the protein sequences suggest different regulatory mechanisms (Supplementary Fig. 1, Supplementary Data 1). Recently it has been found that ToCsm6 autoregulates its activity by cleaving cOA_**4**_ in its CARF domain^22^; however, no information is available for the Csx1 RNase family. To address that in the current study, we solved the atomic structures of SisCsx1 in its apo and cOA_4_ (cOA composed of 4 AMP units) bound forms. We show that cOA_4_ binding activates a cooperative sequential catalytic response in a hexameric SisCsx1 complex and that the RNase activity is switched-off by Ring nucleases, which cleave cOA_4_^23^. Further, a structure-function analysis revealed that the activation by the cyclic molecule triggers specific cleavage of phosphodiester bonds between cytidines.

## Results

### SisCsx1 is a hexameric RNase

**Fig. 1:**
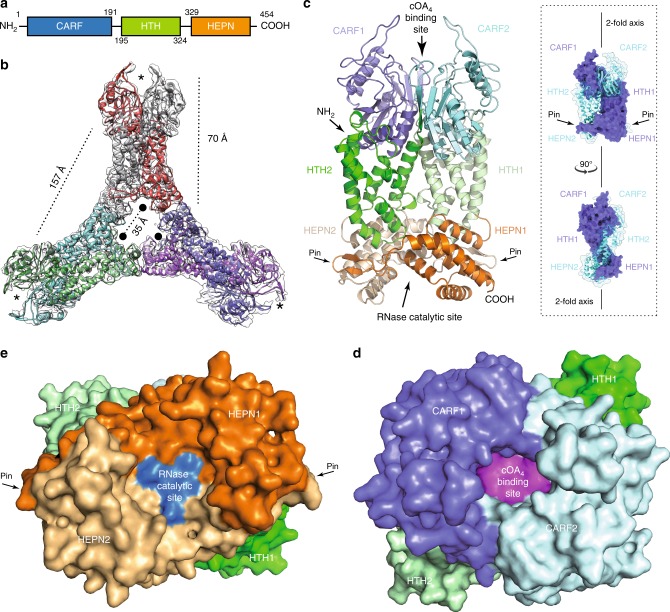
Structure of SisCsx1 endoribonuclease. **a** Domain organization of SisCsx1. **b** Cartoon model of the apo SisCsx1 crystal structure superimposed on the cryoEM map used to solve the phase problem. Each monomer is colored differently. Asterisks indicate the cOA_4_ binding sites and the black circles the catalytic pockets. **c** The monomers of SisCsx1 are twisted around the dimer axis. Detailed view of one of the dimers with the different domains showing the cOA_4_-binding and catalytic pockets. The monomers are shown following the color scheme in panel **a**. A pale tone of the same color is used to differentiate each monomer. **d** Top view of the cOA_4_-binding pocket between the CARF domains colored in magenta. (**e**) Bottom view of the SisCsx1 dimer showing the RNase catalytic site built by the HEPN domains (colored in blue)

SisCsx1, as other members of the Csm6/Csx1 family^5,12^, is composed of an N-terminal CRISPR-Cas-associated Rossmann Fold (CARF) domain connected to an α-helical higher eukaryotes and prokaryotes nucleotide-binding (HEPN) domain by a helix-turn-helix (HTH) domain (Fig. 1a). We expressed SisCsx1 in *S. islandicus*, and the protein was purified as previously described^24^. A SEC-MALLS analysis revealed that SisCsx1 is an oligomer of 300 kDa corresponding to a hexameric assembly (Supplementary Fig. 2). A cryoEM map of the complex at medium resolution (Fig. 1b, Supplementary Fig. 3, Supplementary Table 1) was used as a model to solve the phase problem by molecular replacement in an apo SisCsx1 crystal belonging to the P2_1_ space group (Methods). The Cα backbone built in this map was later combined with an iodine derivative of a better diffracting crystal of SisCsx1 in the space group I2_1_2_1_2_1_ to determine the structure of the apo SisCsx1 to 2.9 Å. Subsequently the apo SisCsx1 structure was used two determine by molecular replacement two SisCsx1 conformations in complex with cOA_4_ at 3.1 and 2.7 Å resolution respectively (Supplementary Table 2, Methods), thus providing the molecular details of the RNase activation by the secondary messenger.

The SisCsx1 monomers are curled around the two-fold axis to form a dimer (Fig. 1c), and three of these dimers oligomerize through their HEPN domains into an equilateral trimer, thus assembling into a hexamer (Fig. 1b). Each dimer contains a cOA_4_ binding site at the vertex of the triangular assembly (Fig. 1d), and a catalytic pocket located 70 Å away inside the triangular oligomer along the two-fold dimer axis (Fig. 1e). The two-fold axis bisects the cOA_4_ and catalytic pockets in the dimer. Hence, the triangular assembly forms two equilateral triangles, one inside the other, with the vertexes formed by the CARF and HEPN domains, building the cOA_4_ binding and catalytic sites in each dimer (Fig. 1b, c).

**Fig. 2:**
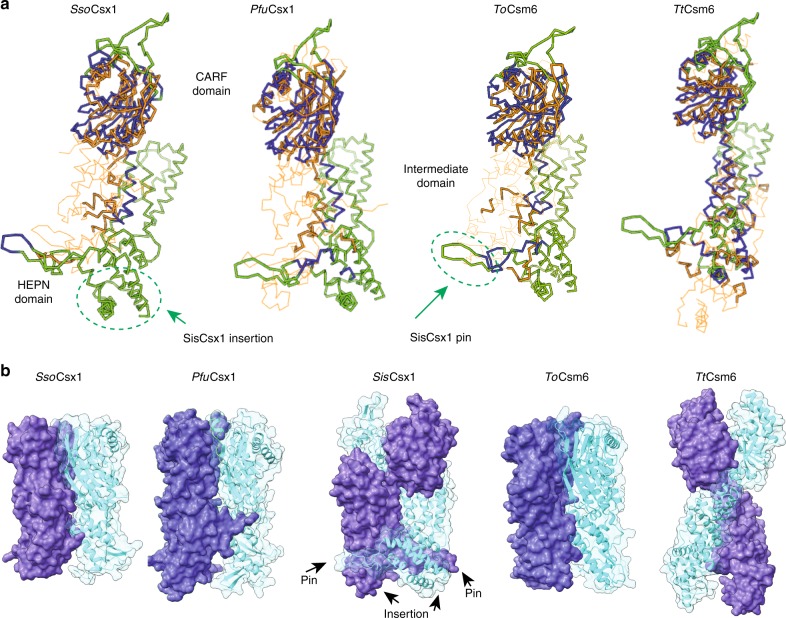
Comparison between type III CRISPR-associated ribonucleases. **a** Structural comparison between SisCsx1 and the dimeric SsoCsx1, PfuCsx1, ToCsm6, TtCsm6. Ribbon representation of the *Sis*Csx1 (green) and PfuCsx1, SsoCsx1 and TtCsm6 (orange) monomers which have been superposed using the DALI server. SsoCsx1 (PDB 2I71) r.m.s.d. to SisCsx1 is 5.1 Å for 184 residues out of 376 with an identity of 20%, for PfuCsx1 (PDB 4EOG) r.m.s.d. to SisCsx1 is 6.1 Å for 200 residues out of the 466 of the protein with an identity of 12%, for ToCsm6 (PDB 6O6S) r.m.s.d. to SisCsx1 is 6.0 Å for 194 residues out of the 433 of the protein with an identity of 15%, while for TtCsm6 (PDB 5FSH) the r.m.s.d. is 5.0 Å for 224 out of the 448 of the protein with an identity of 11%. **b** Comparison of the dimeric arrangements of SsoCsx1, PfuCsx1, ToCsm6, TtCsm6 with SisCsx1

Despite the conservation of the domain architecture between Csm6 and Csx1 RNases, a comparison of SisCsx1 with ToCsm6, TtCsm6, PfuCsx1 and SsoCsx1 reveals large differences in protein sequence (Supplementary Fig. 1) and topology, especially in the HTH and HEPN domains (Fig. 2a, b). Only the CARF domain displays structural conservation between these proteins. These differences determine that in SisCsx1, the monomers are curled around the dimer axis (Fig. 1c, Fig. 2b), while the dimers of the other Csx1 and Csm6 RNases interact along the axis from the CARF to the HEPN domains (Fig. 2b). In addition, the sequence alignment and the superposition reveal a unique insertion region in the HEPN domain of SisCsx1, which is the responsible for the protein hexamerization (Supplementary Fig. 1, Fig. 2a, b). Collectively, these observations suggest that while cOA_4_ binding is conserved, the mode of RNase regulation may be different.

### SisCsx1-cOA_4_ complex displays different conformations

**Fig. 3:**
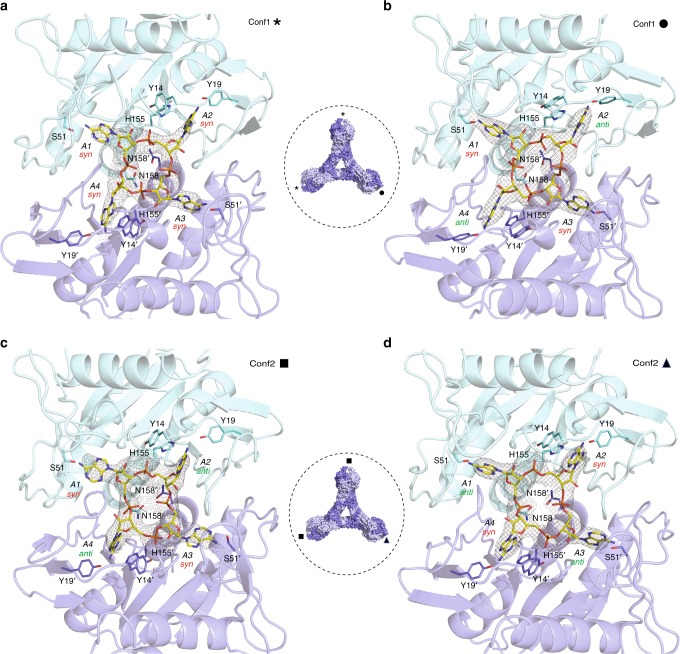
cOA_4_ configurations in the binding pockets. **a** Detailed view of the cOA_4_ binding site in conf1 including its interactions with key side chains. This configuration is observed in two of the binding pockets of the hexamer (indicated with an asterisk). **b** The third pocket in conf1 exhibits a different configuration of cOA_4_ (indicated with a black circle). **c** Detailed view of cOA_**4**_ binding site in conf2 including key side chain. This configuration is visualized in two of the binding pockets of the hexamer (indicated with a black square). **d** The conformation of cOA_4_ is also different in the third binding pocket in conf2 (indicated with a black triangle). The 2mFo-DFc electron density maps in panels A-D are displayed at 1.1 σ contour. Views of the omit maps of **a**–**d** panels are provided in Supplementary Fig. 7B

The SisCsx1-cOA_4_ complex structure provides evidence of how the cOA_4_ activator binding to the CARF domain triggers RNA degradation. We synthesized cOA_4_ using purified Type III-B SisCmr−α complex (Supplementary Fig. 4), and observed that the binding of cOA_4_ at room temperature, barely activates SisCsx1, while heating the reaction mixture triggered full RNase activity (Supplementary Fig. 5a). As with other HEPN domain nucleases, SisCsx1 does not require metal cofactors for its activity (Supplementary Fig. 5b). To explore these differences, we crystallized the SisCsx1-cOA_4_ complex by soaking the compound in crystals of the apo SisCsx1 at 25 °C, and we also co-crystallized the complex after heating up the mixture of cOA_4_ with the isolated SisCsx1 to monitor possible conformational changes that could elicit the RNase activity by the presence of cOA_4_. The different crystallization procedures yielded the same type of crystals (Supplementary Table 2), which displayed two different conformations of cOA_4_ (conf1 and conf2) in the binding pocket, along with rearrangements of the protein moiety (Fig. 3, Supplementary Fig. 6, Supplementary movie 1, 2).

The dimeric assembly of SisCsx1 is essential for the formation of the cOA_4_ binding and catalytic pockets by the CARF and HEPN domains. The curled arrangement of the monomers positions the CARF domain of one monomer on top of the HTH domain of the other, while the regions spanning residues 405–427 in the HEPN domains form two “pins” that clip each monomer between the HTH and the HEPN domains (Fig. 1c–e, Fig. 2b). The cOA_4_ binding cleft is formed by the loops connecting several secondary structure elements of the CARF domains, including residues in loops spanning residues 6–18, 28–30, 47–54, 92–100, 153–158 and 178–188. The conformational changes upon cOA_4_ binding induce a reshaping of the loops, so that the oval cleft observed in the apo structure shifts to form a cruciform pocket, in which the cOA_4_ is bound (Fig. 3, Supplementary movie 1). This reconfiguration of the cOA_4_ binding pocket promotes a change in the electrostatic potential of the cavity turning the neutral potential observed in the apo structure to the polar cleft observed in conf1 and conf2 (Supplementary Fig. 7A).

### A conf1-conf2 transition triggers RNase activity

Each Csx1 monomer hosts a pair of adenines of cOA_4_, A1-A2 and A3-A4 (Fig. 3). A comparison of the cOA_4_ conformations in conf1 and conf2 revealed that the molecule undergoes a conformational change in the binding pockets of the hexamer (Fig. 3, Supplementary movie 1). In the conf1 configuration, cOA_4_ displays a “wrinkled” conformation of the cyclic compound with all bases in a *syn* or near to *syn* arrangement in two of the second messenger binding pockets (Fig. 3a, Supplementary Fig. 6). The A1 base makes polar interactions with the main chain of S51 and A1 ribose 2-OH interacts with the main chain of D10. A2 is stabilized in the *syn* configuration by polar contacts with the main chains of S15 and F29. All contacts in the A3, A4 pair are made by residues in the second monomer where these nucleotides are located. The A3 base is stabilized in a near *syn* configuration by interactions with the side chain of S51′ as well as with the main chains of S51′ and S98′ while A3 ribose 2-OH contacts with the side chain of Y14′. A3 phosphate interacts with G182. The A4 phosphate contacts the G156′ amide thanks to N158, which arranges the loop by contacting with the main chain of T154′ and G156′ to promote that interaction. Finally, the A4 base is stabilized in the *syn* configuration by polar contacts with the side chain of Y19′ and the main chain of S15′ and F29′. Interestingly, A4 phosphate is stabilized by contacting with the side chains of N158 and N158′ through a water molecule. Multiple polar and hydrophobic contacts with the main and side chains of several residues in the cleft contribute to accommodate the cOA_4_ molecule. However, in the third binding site, cOA_4_ displays an alternate configuration *syn* and *anti*, due to a conformational change to the *anti* configuration in A2 and A4 by stabilizing their contacts with Y19 and Y19′, respectively (Fig. 3a, b, Supplementary Fig. 6).

**Fig. 4:**
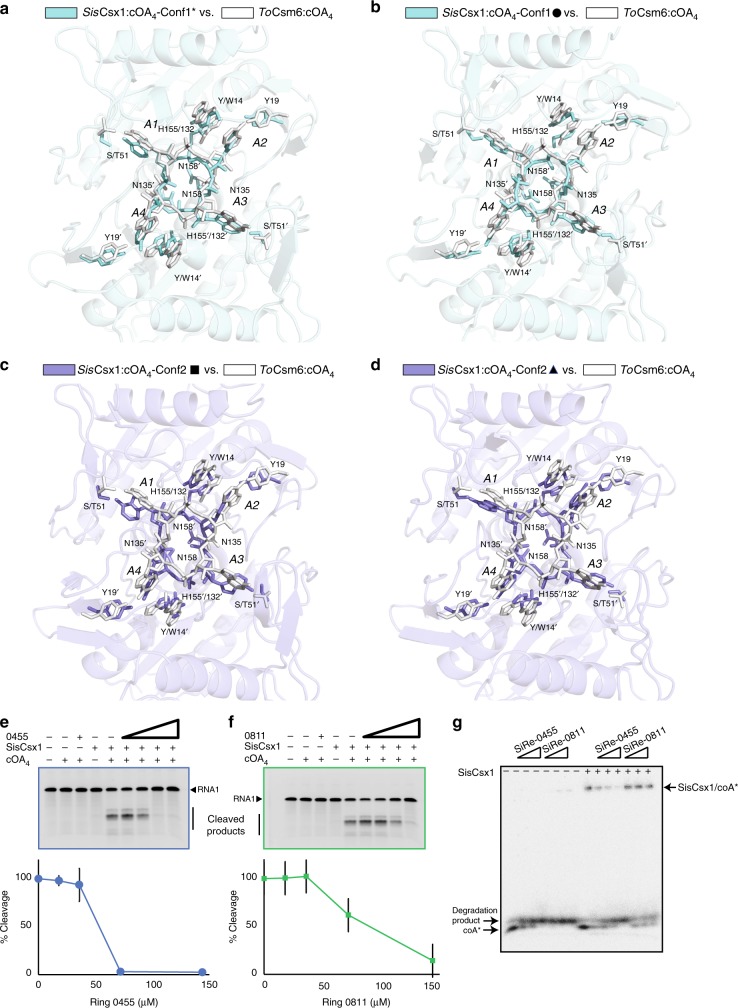
Comparison of SisCsx1:cOA_4_ and ToCsm6:cOA_4_ CARF domains and RNase deactivation. Detailed view superimposition at the CARF domains of SiScsx1:cOA_4_ complex in Conf1 asterisk (**a**) or Conf1 solid circle (**b**) vs. ToCsm6:cOA_4_. Detailed view superimposition at the CARF domains of SiScsx1:cOA_4_ complex in Conf2 solid square (**c**) or Conformation 2 solid triangle (**d**) vs. ToCsm6:cOA_4_. *S. islandicus* Ring nucleases 0455 (**e**) and 0811 (**f**) deactivate the RNase activity of SisCsx1. Increasing concentrations of both Ring nucleases from 10 nM to 150 nM were incubated with 25 nM of cOA_4_ at 70 °C for 30 min followed by addition of 18 nM of SisCsx1 and 2.5 μΜ of RNA1 and further incubation at 70 °C for 5 min. The reactions were then separated using 15% Novex TBE-urea gel (Invitrogen). **g** Cleavage of cOA_4_ by Ring nucleases 0455 and 0811. On the left side of the gel cOA_4_ is incubated with Ring nucleases 0455 and 0811. The cyclic compound migrates faster than the linear product generated by the ring nucleases. On the right side of the gel SisCsx1 has been included in the reaction showing that it does not cleave cOA_4_ and protects its degradation by the ring nucleases. Each experiment for the **e**, **f**, **g** panels has been repeated at least three times. The error bars represent the s.d. Source data are provided as a Source Data file for 4E-G

The disposition of cOA_4_ in conf2 reveals a rearrangement of the cyclic compound, whose configuration coincides again in two of the three binding pockets (Fig. 3c, d, Supplementary Fig. 6). An inversion of the phosphate groups forces the P = O bonds of A1 and A3 towards the bottom of the pocket, inducing the changes from *syn* to the *anti* conformation of the A2 and A4 nucleotides, thus transiting from the “wrinkled” conformation observed in conf1 to a more symmetrical arrangement in conf2. This change in the phosphate backbone and base configuration is supported by the Fo-Fc omit maps of the cyclic compound and is accompanied by new polar and hydrophobic interactions in the binding site (Supplementary Fig. 7b-c, Supplementary movie 1). The phosphate inversion in cOA_4_ is stabilized in both cases by hydrogen bonds of the phosphate groups of A1 and A3 with N158 and N158′ through a water molecule. A rotamer change of H155 in both monomers allows the polar interaction of the imidazole group with the A2 and A4 bases in each monomer, thus positioning the cyclic compound into the cruciform pocket. In addition, A2 and A4 phosphates are stabilized by the side chains of Y14 and Y14′. Finally, the *anti* conformation of A2 and A4 is stabilized by interactions of the base with the main chain of S15, S15′, F29′, and V180′ and the OH of Y19′, while the *syn* configurations observed for A1 and A3 are stabilized by polar interactions with the main chain of D10, D10′, S51, S51′, and S98 and hydrophobic contacts with M181 side chain in both monomers. The alternate configuration observed in the third binding pocket is accompanied by a reshaping of the polar and hydrophobic interactions in the binding site (Fig. 3c, d, Fig. 4b–d, Supplementary Fig. 6).

### Comparison of cOA_4_ binding by ToCsm6 and SisCsx1

The amino acids involved in the cOA_4_ binding are well-conserved between the CARF domains of Csx1 and Csm6 ribonucleases. These include SisCsx1 residues Y14, Y19, S51, T154, H155, G156, N158, while the hydrophobic V180 and M181 could be substituted by other non-polar residues (Supplementary Fig. 1). A recent analysis of the ToCsm6-cOA_4_ complex shows that upon binding, the cyclic molecule is cleaved by the CARF domain, thus deactivating its RNase activity^22^. However, W14 and H132, which have been identified as important residues in the CARF domain of ToCsm6 for cOA_4_ cleavage, are not well conserved among the Csm6/Csx1 proteins. A genomic analysis of 294 prokaryotic genomes shows that these two residues are present in less than 10% of the Csm6 proteins and they are frequently replaced by Y and S respectively (Supplementary Data 1). In the case of the Csx1 family only 5% of the proteins encode these residues in the equivalent positions, the H residue is commonly found while W is substituted by other amino acids, generally by Y, as observed in SisCsx1 (Supplementary Fig. 1, Supplementary Data 1). A superposition of the ToCsm6 and SisCsx1 structures shows that the conformations of the CARF domain and the cyclic molecule are different (Fig. 4a–d), specially because cOA_4_ is not cleaved in the SisCsx1 structures (Fig. 3, Supplementary Fig. 7B-C). ToCsm6 cOA_4_ degradation involves W14, H132 and N135 residues, corresponding to Y14, H155 and N158 in SisCsx1 (Fig. 4a–d), consequently, the key W14 is substituted by Y14 in SisCsx1 altering the possible catalytic configuration. In addition, the configuration of N135 side chain, which has been linked to the cleavage of cOA_4_^22^, can be found pointing inwards (non-cleaved cOA_4_) or outwards (cleaved cOA_4_) of the cyclic oligoadenylate ring. In contrast, we observed that N158 in SisCsx1, is pointing inwards in the apo, conf1 and conf2 structures (Fig. 4a–d). Overall, these observations indicate that the CARF domain of SisCsx1 cannot cleave cOA_4_.

### SisCsx1 RNase activation by cOA_4_ is regulated by Ring nucleases

To elucidate whether SisCsx1 can cleave cOA_4_, we performed a cleavage assay of the cyclic molecule in the presence and absence of Ring nucleases, which have been shown to deactivate the cOA_4_ -stimulated RNAse activity of the Csx1 family members via degradation of the cyclic molecule^23^. We tested whether SisCsx1 could cleave cOA_4_ or whether its activation by cOA_4_ was affected by the presence of Ring nucleases (Fig. 4e–g). No cleavage of the cyclic molecule by SisCsx1 could be observed in the absence of Ring nucleases, as it was equally observed for the dimeric SsoCsx1^23^. However, a substantial decrease of SisCsx1 RNase activity could be observed in the presence of Ring nucleases, thereby endorsing the deactivating effect caused by the action of these enzymes on the cyclic compound^23^. Consequently, the lack of conservation of key amino acids for catalysis (ToCsm6 W14 and H132) in the Csm6 family and the absence of cOA_4_ cleavage in SisCsx1, suggest that different RNase regulatory mechanisms are employed by other members of the Csm6/Csx1 families.

### SisCsx1 activation by cOA_4_ displays cooperativity

SisCsx1 binding of cOA_4_ is cooperative with a Hill coefficient n, of 2 and a *K*_*d*_ of ~18 nM (Supplementary Fig. 8A). Single mutants in the CARF domain such as N158A, H155 A and S51A are still able to display detectable RNase activity and a double mutation H155D/N158D is needed to abrogate RNase activity (Supplementary Fig. 8B). The shape of the pocket and the conformational change upon second messenger binding is reminiscent of a “tippy tippy tap” paper toy, i.e., the configuration of the binding pocket is coupled with conformational changes in the cOA_4_ molecule to accommodate the compound. Collectively, these findings indicate that the configuration of cOA_4_ undergoes conformational changes between conf1 and conf2, which are transmitted to the HTH domain for RNase activation (Supplementary movie 2).

**Fig. 5:**
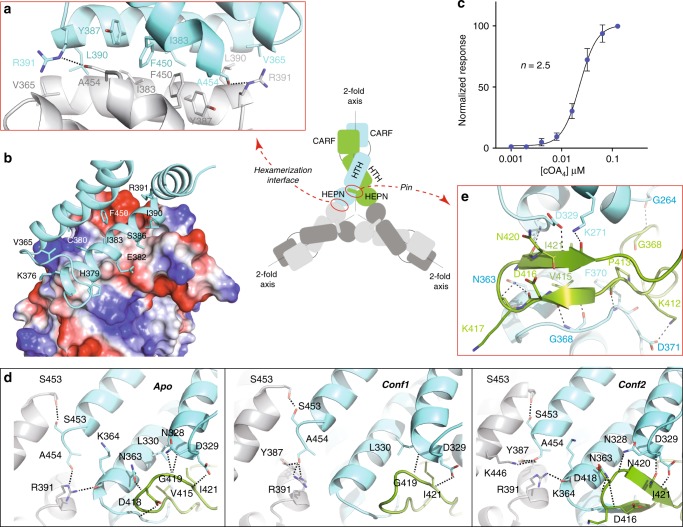
Molecular basis of SisCsx1 cooperativity in ssRNA degradation. **a** Detailed view of the SisCsx1 helices building the hexamer interface. The sketch shows the domain organization of one of the SisCsx1 dimers in the hexameric arrangement. **b** Electrostatic potential of the interaction surface between the HEPN domains. **c** SisCsx1 RNase activity displays a cooperative behavior in the presence of increasing concentrations of cOA_4_. The experiment was repeated 3 times, the error bars represent the s.d. (Supplementary Fig. 8D). **d** Detailed view of the oligomerization interface in the apo, conf1 and conf2 structures showing the interplay of the hydrogen bond network between R391, Y387, K446, A453, and K364. **e** Detailed view of the pin of one of the HEPN domains (residues 405–427), which inserts in the neighbouring monomer. This feature is conserved in all the catalytic sites of the assembly forming a staggered arrangement (See also Supplementary movie 3-4)

The structure of SisCsx1 and cOA_4_ binding data strongly suggested that the endoribonuclease activation by the cyclic compound is cooperative. The SisCsx1 hexamer is built because of a split helical insertion (V365-N400) and the last helix at the C-terminus (D445-A454) in the HEPN domain (Fig. 5, Supplementary Fig. 1). The α-helix spanning residues 375-391, establishes a series of contacts with the same segment in the adjacent monomer with the helices inversely oriented (Fig. 5a), and a well-conserved cysteine bridge (C361-C380) positions the hexamerization helix. The helices fit into each other and the central interaction surface is mainly hydrophobic. The hydrophobic interface is flanked by several polar interactions (Fig. 5b). To understand how catalysis works upon cOA_4_ binding, we performed activity experiments, which demonstrated that SisCsx1 displays cooperative catalysis (Fig. 5c, Supplementary Fig. 8d). The cleavage of a ssRNA substrate was quantified in the presence of increasing amounts of cOA_4_. The sigmoidal curve displayed a Hill coefficient n, of 2.5, indicating a cOA_4_ dependent cooperativity to catalyze phosphodiester hydrolysis of the ssRNA substrate. The hydrogen bond pattern of the interaction surfaces between the HEPN domains of the hexamer is different in the apo, conf1 and conf2 structures (Fig. 5d), revealing that cOA_4_ binding induces a remodeling of the interactions in the catalytic domains. While the central hydrophobic interfaces are very similar in the three conformations, an interchangeability of the hydrogen bonding scheme of R391, Y387, K446, A453 and K364 could be observed between the apo, conf1 and conf2 structures (Fig. 5d, Supplementary Table 3, Supplementary movie 3). These interactions occurring at the C-terminus of each monomer appear to provide a signaling pathway within the HEPN domains of the hexamer.

Interestingly, this region is in the neighborhood of the pin extension from another monomer, which fits snugly between the HEPN and HTH domains, providing a staggered interaction of the catalytic sites in the hexamer (Fig. 1e). This arrangement of the hexamer provides the scaffolding supporting a sequential cooperativity mechanism transmitting the cOA_4_ binding signal from the CARF through the HTH (Supplementary movie 2) domains to the catalytic sites in the different HEPN domains (Supplementary movie 4) through the hexamer oligomerization interface (Supplementary movie 3). To test this observation, we designed the I383A/L390D/R391A mutant, which disrupts key interactions for oligomerization in the helical insertion (residues 375-391) without compromising dimer assembly (Supplementary Fig. 2). The I383A/L390D/R391A mutant displayed cOA_4_ binding (Supplementary Fig. 8a); however, its RNase activity is severely affected indicating that the hexamer is essential for ssRNA decay (Supplementary Fig. 8c).

The high RNase activity (Supplementary Fig. 5a) and the changes observed in the HEPN interfaces, suggest that upon cOA_4_ binding, the change of conformation between conf1 and conf2 (Fig. 2) would be coupled with the full activation of the RNase activity by signaling the conformational change through the HTH and the HEPN pins (Supplementary movie 4), thus remodeling the contacts between the HEPN hexamer interfaces (Fig. 5, Supplementary movie 3). Collectively, the analysis indicates that the interplay of the interactions in the HEPN domain interfaces is a consequence of the intramolecular signaling of cOA_4_ binding, which is propagated through the hexamer to induce RNase activity in the catalytic sites.

### SisCsx1 catalytic center

**Fig. 6:**
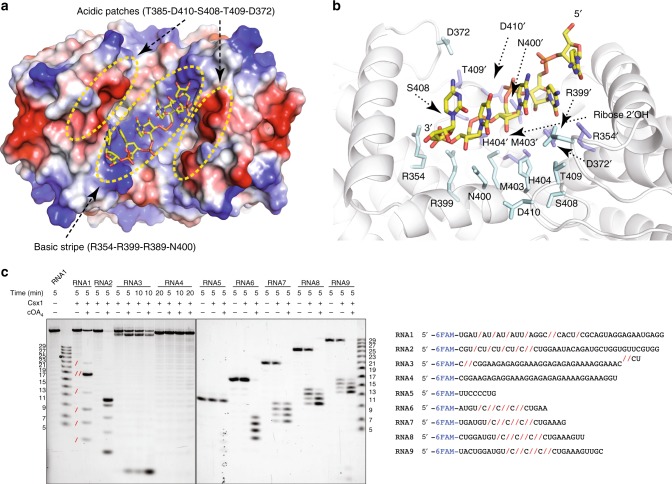
Catalytic pocket of SisCsx1 in the HEPN domains and cleavage specificity. **a** Docking of polyC ssRNA (stick model) into the catalytic pocket of a SisCsx1 dimer. **b** The docked polyC molecule and the positioning of the 2′-OH of ribose with respect to one of the H404 residues for the initiation of phosphodiester hydrolysis. Residues from each monomer are displayed in cyan and blue respectively. The residues in the later one is labelled with ´. **c** SisCsx1 shows a strong preference for the hydrolysis of phosphodiester bonds between 5′-C–C-3′ located in the central section of ssRNA, as determined by activity assays using RNA1–RNA4. The substrates are at least 9–12-nt-long, as determined by activity assays with RNA5–RNA9. Red slashes in the gel and ssRNA substrates (below the gel), the cleaved bond. The experiment has been repeated three times. Source data are provided as a Source Data file for Fig. 6c

The active center cleft comprises R354, D372, R389, R399, N400, M403, H404, T409, and D410 residues (Supplementary Fig. 1, Fig. 6a, b). The SisCsx1 dimeric arrangement results in a symmetric placement of these amino acids, creating a diagonal electropositive stripe flanked by two electronegative regions on each side (Fig. 6a). Changes in the interfaces of HEPN domains observed in the apo, conf1, and conf2 structures induce subtle conformational differences in the side chains of these residues (Supplementary movie 5). In fact, these rearrangements appear to be responsible for the RNase activation. We attempted to co-crystallize SisCsx1 with an excess of cOA_4_ or ssRNA, but we were unable to visualize the cyclic compound or the oligonucleotide in the active site in the HEPN domain. However, we observed two additional densities from sulfate ions present in the crystallization solution associated with R354 and R354′ in the catalytic pocket (Supplementary Fig. 9A). This finding, together with our mutational analysis (Supplementary Fig. 8C) and the electrostatic potential of the catalytic site, offered some hints on orientation of the ssRNA phosphate backbone in the active center. To understand the mechanism of phosphodiester hydrolysis, we utilized the identified key residues in an information-driven flexible docking modeling approach^25,26^ of a polyC oligonucleotide in the catalytic pocket (Fig. 6a). As expected, the docking results revealed a ssRNA backbone located on the diagonal electropositive stripe, with R354, R399, R389, N400 in possible contact distances of the phosphate groups (Fig. 6a, b). In the model, the bases of the ssRNA can interact with the electronegative patches in the upper left and lower right corners of the catalytic cleft where T385, D410, S408, T409, and D372 are located (Fig. 6a, b). The position of the phosphate backbone places one of the phosphodiester bonds between the H404 and H404′ in the center of the cavity, suggesting that these residues are directly involved in catalysis. This conclusion is supported by the observation that the H404A mutation abolishes cleavage (Supplementary Fig. 8C) and the abrogation of SisCsx1 activity at high pH (Supplementary Fig. 5C). Histidine is a residue conducive for phosphodiester catalysis as it can accept and donate protons since the pKa value of histidine is close to neutral. Because of the dual role of histidine in catalysis and depending on whether the ssRNA enters the symmetric catalytic site with a 5′-3′ or 3′-5′ polarity, one of the H404 residues would be positioned to activate the 2′-OH of the ribose, thus initiating the cleavage reaction. The transition state could be stabilized by R399 or N400, which are positioned in the vicinity of the H404.

Some similitudes between SisCsx1 and another RNases with histidines in their catalytic centers can be found. An example is the dimeric RNase L, an interferon activated RNase, which contains two histidines in the active center. However, phosphodiester catalysis by RNase L supposedly involves only one of them^27^. In SisCsx1, the two histidines are close and the movements of the HEPN domains observed in the different structures do not suggest large rearrangements in the active site after cOA_4_ binding (Supplementary movie 5) as observed in RNase L, suggesting that both histidines are involved in catalysis. The proposed mechanism more closely resembles that of RNase A, a classical ssRNA endonuclease, which also contains two histidines in the catalytic site but lacks the two-fold symmetry observed in the SisCsx1 dimer. These histidines alternate their role as proton donor and acceptor during catalysis^28^. Although, further analysis will be needed to fully decipher SisCsx1 ssRNA phosphodiester hydrolysis, the current evidence suggests that SisCsx1 catalytic center would allow phosphodiester hydrolysis independently of the ssRNA substrate polarity in the pocket.

### SisCsx1 cleaves phosphodiester bonds between cytidines

**Fig. 7:**
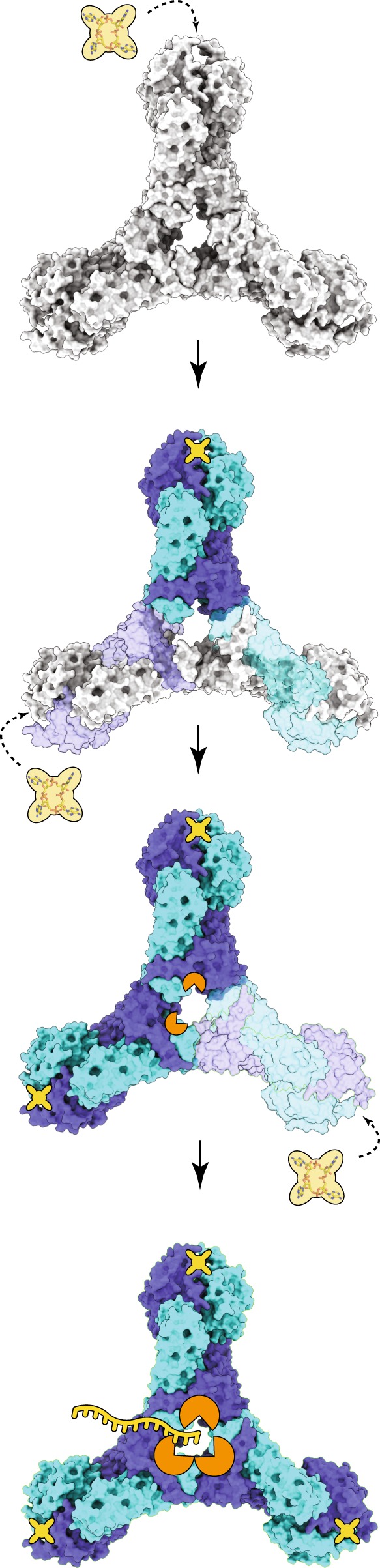
Model for SisCsx1 RNase catalysis activation by cOA_4_. The binding of cOA_4_ (tetralobal shape) by the CARF domains fully activates the first dimer (strong blue and strong sky blue) and induces a conformational change through the HTH to the HEPN domains of two adjacent monomers (light blue and light sky blue) favoring cOA_4_ binding in any of their available cOA_4_ binding sites. The binding of the second cOA_4_ molecule fully activates the second dimer (strong blue and strong sky blue), promoting some initial RNase activity and triggering conformational changes in the two monomers of the third dimer (light blue and light sky blue) leading to the binding of the third cOA_4_ molecule and the full RNase activation

Some endoribonucleases can catalyze reactions involving RNA molecules containing specific sequences, structures or sequences within a specific structure providing tools for RNA manipulation. However, a possible SisCsx1 cleavage specificity was ambiguous^24^. In contrast with previous suppositions, we observed that SisCsx1 exhibits a strong preference for cleaving substrates with phosphodiester bonds between 5′-C–C-3′, and with a minor and very low efficiency ones with phosphodiester bonds between 5′-U-C-3′ and 5′-U-A-3′ (Fig. 6c). Such cleavage preference likely arises from the putative contacts between the cytosine base and the acidic regions of the pocket (Fig. 6a, b). Further activity experiments with different ssRNA substrates revealed that the cleavage occurs in the central part of the oligonucleotide, with the efficiency of the otherwise preferential 5′-C–C-3′ cleavage greatly diminished when the phosphodiester bond between cytidines is located at the 5′- or 3′-end of the substrate molecule (Fig. 6c). The cleavage assay also reveals that the ssRNA must be at least 12nt long to observe cleavage. As the distance between the catalytic centres is around 35 Å, a ssRNA substrate between 10-12 nucleotides could interact with two catalytic sites, suggesting that they may work on the same RNA molecule simultaneously, which would agree with the observed cooperativity. Based on the data presented in this manuscript, we propose a sequential cooperativity model for SisCsx1 RNase. In this model, cOA_4_ binding subsequently signals to the monomers in the other two dimers of the hexamer to facilitate binding of other cOA_4_ molecules promoting full activation of the RNase activity (Fig. 7).

## Discussion

In this study, we present mechanistic evidence regarding SisCsx1 ssRNA cleavage activation by cOA_4_, thereby showing the molecular basis of how this second messenger acts on members of the Csx RNase family to trigger a massive RNA decay. Our work shows that the SisCsx1 oligomer displays positive sequential cooperativity to hydrolyze phosphodiester bonds after binding cOA_4_, following the Koshland, Némethy and Filmer (KNF) model (Fig. 7). The binding of the cyclic oligoadenylate promotes the conformational switch on the other subunits by decreasing the energy making the conformational change thermodynamically favourable; hence, facilitating that the rest of the SisCsx1 molecules undergo the conformational change allowing binding. Our work shows that full SisCsx1 activation is achieved after a transition of cOA_4_ configuration upon binding, thus triggering a large ssRNA decay to stop infections. Although, we could not unveil the reason of the strong preference displayed by SisCsx1 to cut C–C phosphodiesters in ssRNA, we speculate that this cleavage preference could be related to their abundance in sequences that infect this type of bacteria or in mRNAs that could code for proteins that avoid the cell to enter quiescence.

There are obvious differences between the activation mechanism of ToCsm6 and SisCsx1. To rationalise the distinct type III RNase regulation by cOA_4_, we performed a genomic analysis using a large number of prokaryotic genomes (Supplementary Data 1). Our study shows that Csm6 (Type III-A) proteins seem to be preferentially dimers, as a BLAST search found no Csm6 containing the SisCsx1 insertion region in their HEPN domain. In addition, only few Csm6 genes display the residues (W14 and H132) involved in cOA_4_ cleavage by the CARF domain of ToCsm6, suggesting that the majority of Csm6 proteins may not cleave cOA_4_ to autoregulate their activity. An example is TtCsm6 where these residues are substituted by Y and S respectively (Supplementary Fig. 1, Supplementary Data 1), indicating that cleavage of cOA_4_ by the CARF domain of the RNase may not be a general mechanism in the Csm6 family. Among the genomes containing Csx1 (III-B, III-C, and III-D), species belonging to the *Sulfolobus, Sulfurisphaera, Acidianus and Metallosphaera* geni, display the oligomerization insertion, and therefore are most likely hexamers, while the rest lack this sequence and seem to be dimers. Almost all Csx1 proteins, including both dimers and hexamers, do not contain the W14 and H132 residue combination involved in cleavage of the cOA_4_ by the CARF domain of ToCsm6. In addition, it has been shown that SsoCsx1 (dimer) does not cleave cOA_4_^23^, similarly to SisCsx1 (hexamer) (Fig. 4g). Both species contain Ring nuclease genes, suggesting that organisms encoding both Csx1 and Ring nucleases do not seem to cleave cyclic oligoadenylates using the CARF domain of the RNase, but rather use the Ring nucleases to deactivate Csx1 RNase activity. Collectively, our analysis indicates that the differences observed between both Csm6 and Csx1 RNases and their regulatory mechanisms, represent the large diversity of strategies developed during evolution to adapt the heterogeneous CRISPR-Cas Type III systems to the habitats of these organisms. Given the large number of prokaryotes containing Type III systems new regulatory mechanisms could be found in future studies.

CRISPR-Cas systems have often been considered as a prokaryotic immune system that fight viruses and other mobile genetic elements. An analysis of how the Type III CRISPR-Cas systems address viral infection, in conjunction with the presented mechanistic analysis of SisCsx1 ssRNA degradation, is strikingly reminiscent of the 2,5-oligoadenylate synthetase (OAS)/RNase L system, one of the principal IFN antiviral pathways^29^, which involves the activation of RNase L, an ubiquitous cellular endoribonuclease^27^. Although there are obvious differences with the Type III CRISPR-Cas, the general architecture of the defense response exhibits a similar mechanistic organization: after an initial step during which the viral genetic material is sensed as an invader (by Type III Csm/Cmr complexes), a second messenger molecule is produced (cOA) to activate an oligomeric RNase (Csm6/Csx1), which (at least in the case of Csx1) specifically cleaves ssRNAs in the cell. Collectively, all these resemblances suggest that CRISPR-Cas Type III systems may be prokaryotic ancestors of the more elaborated innate immunity pathways in eukaryotic systems.

The activation of the SisCsx1 RNase catalytic activity in the presence of cOA_4_, together with the specific cleavage between cytidine sequences and the possibility to inhibit ssRNA decay using Ring nucleases (Fig. 4e, f), suggest that SisCsx1 could be employed as a modular switch in synthetic biology applications, thus controlling gene expression by regulating mRNA decay. Further, the RNase activity could be used in nucleic acid detection, as in the recently developed SHERLOCK system as its “cousin” Csm6^30^.

## Methods

### Strains and growth conditions

*Sulfolobus islandicus* strains employed in this work included the genetic hosts E233S1 (Δ*pyrEF*ΔlacS), E233 (Δ*pyrEF*)^31^ and Δcsx1Δα, a *csx1* deletion strain lacking the* cmr-α* gene cassette derived from E233 (Supplementary Table 4). The strains were grown in SCV medium (0.2% sucrose, 0.2% Casamino acids, 1% vitamin solution, and basic salts) at 78 °C. If required, uracil was supplemented to 20 μg/ml. Transformation of Sulfolobus cells was performed by electroporation as previously described^32^.

### Construction of *S. islandicusΔcsx1Δα*

The *Δcsx1Δα* mutant was constructed using the CRISPR-based genome-editing method^33^. A protospacer was identified in the *csx1* gene with which two oligonucleotides were designed (csx1spacerI–F and csx1spacerI-R) and annealing of the two oligonucleotides gave the spacer fragment, which was cloned into pSe-RP^9^, giving pAC-csx1-S1. Homologous arm of the *csx1* deletant allele was generated by SOE-PCR using csx1-F-SalI, csx1-SOE-R, csx1-SOE-F, and csx1-R-NotI. Insertion of the homologous arm into pAC-csx1-S1 yielded pGE-csx1 (Supplementary Tables 4–5-6). The plasmid was introduced into *Δα*, a *cmr-α* deletant strain derived from E233^11^ by transformation, giving *Δcsx1Δα*. The genotype of the mutant was confirmed by PCR amplification of the deletant allele of *csx1* and sequencing of the resulting PCR fragment. PCR was conducted using Phanta Max Super Fidelity DNA polymerase (Vazyme).

### Construction of expression plasmids

Six new substitutions were designed for the *S. islandicus* Csx1, namely H404A, S51A, N158A, I383A/L390D/R391A, H155A and H155D/N158D (Supplementary Table 6). The Csx1 expression plasmid of Csx1, pSe-Csx1^24^, was employed as the template to generate the designed substitutions using In-Fusion^®^ HD cloning kit (Takara Bio USA, Inc), and the mutagenesis was conducted using the primers listed in Supplementary Table 6, following the instruction of the manufacturer. All primers (Supplementary Table 6) were synthesized by the Integrated DNA Technology (IDT, USA). The sequences of the wild-type *csx1* gene and the designed mutations were verified by DNA sequencing of the constructed expression plasmids at MacroGen Europe (Amsterdam, Netherlands). Sequences encoding for the Ring-0455 and Ring-0811 were synthesized by IDT. The genes were then cloned by In-Fusion HD Cloning Plus (Tanaka) into pET-21.

### Purification of Csx1 and its protein variants from *S. islandicus*

His-tagged Csx1 and its mutated derivatives were over-expressed in, and purified from, *S. islandicus* cells as previously described^24^. Each expression plasmid was used to transform strain *Δcsx1Δα*. The resultant transformants were grown in SCV medium at 78 °C until A_600_ = 0.6–0.8. Then, 50 ml of the culture was transferred to 1 l of a prewarmed ACV medium (similar to SCV medium but with 0.1% arabinose instead of 0.2% sucrose) to induce the production of the Csx1 protein or its variants. When the cell density was reached A600 = 0.8, the cells were harvested by centrifugation. The cell pellet was re-suspended in buffer A (20 mM HEPES pH 7.5, 30 mM imidazole, and 500 mM NaCl). Cell envelope was disrupted by using French press. After removing cell debris by centrifugation (10,000 × *g*, 30 min at 4 °C), the resulting cell extract was loaded on a 1 ml HisTrap HP column (GE Healthcare). After washing with 30 ml of Buffer A, the His-tagged protein was purified by elution with a linear gradient of imidazole (30–500 mM), which was generated by mixing Buffer A and Buffer B (20 mM HEPES pH 7.5, 500 mM imidazole, and 500 mM NaCl). The elution fractions containing Csx1 proteins were pooled, concentrated and further purified by size exclusion chromatography (SEC) in Buffer C (20 mM Tris-HCl pH 8.0, and 300 mM NaCl) using a Superdex 200 Hiload column (GE Healthcare). The quality of Csx1 proteins was evaluated by SDS-PAGE. The Csx1-containing fractions were pooled together and stored at 4 °C for further study.

### Purification of Ring nucleases

Proteins purifications of pET21-Ring-0455 and pET21-Ring-0811 were expressed and purified from *E. coli* BL21 star (DE3) cells. Cells were grown in LB media containing 50 µg/ml-1 ampicillin at 37 °C until and OD 600 of 0.8 and the expression was induced by adding1 mM of isopropyl β-D-1-thiogalactopyranoside (IPTG) at 16 °C overnight. The cells were harvested and re-suspended in lysis buffer [50 mM HEPES pH 7.5, 500 mM NaCl, 10 mM Imidazole, 1 mM MgSO4, 0.5 mM TCEP, Benzonase]. Cells were lysed by sonication for 10 min with 10 s on and 10 s off cycle. Cell debris and insoluble particles were removed by centrifugation. The supernatant was loaded onto a 5 ml Crude HisTrap column (GE Healthcare) equilibrated in buffer A (50 mM HEPES pH 7.5, 500 mM NaCl, 10 mM Imidazole, 0.5 mM TCEP). The elution was performed by a step gradient of buffer B (buffer A plus 1 M imidazole). Enriched protein fractions and applied onto a 5 ml HiTrap Heparin HP column (GE Healthcare) equilibrated with buffer A2 (50 mM HEPES pH 7.5, 100 mM NaCl, 0.5 mM TCEP). The protein was eluted with a linear gradient of 0–100% buffer B2 (50 mM HEPES pH 7.5, 2 M NaCl, 0.5 mM TCEP). Protein-rich fractions were loaded onto a HiLoad 16/60 75 Superdex column (GE Healthcare) equilibrated in buffer G (50 mM HEPES pH 7.5, 100 mM NaCl, 0.5 mM TCEP). The protein peaks were concentrated (using 10 kDa MWCO Centriprep Amicon Ultra devices), flash-frozen in liquid nitrogen and stored at −80 °C.

### Synthesis of cyclic oligoadenylates (cOAs) by Cmr-α-RNP

Synthesis of cyclic oligoadenylates (cOAs) by the Cmr-α-RNP from *S. islandicus* Rey15A was performed as described^19^. Briefly, a reaction mixture containing 20 mM MES pH 6.0, 5 mM MnCl_2_, 5 mM DTT, 100 μM ATP, 40 nM Cmr-α-RNP, and 200 nM SS1-46 target RNA was incubated at 70 °C for 30 min. The reaction was stopped by chilling on ice.

### Analysis of cOA alpha–ATP reaction products by ESI-MS

Products of COA alpha reaction with ATP were analyzed by electrospray ionization mass spectrometry (ESI-MS) coupled with liquid chromatography (LC). ESI-MS was performed in a negative mode using MicrTOF QII mass spectrometer (Bruker) with on-line Ultimate 3000 HPLC system (Dionex). Reaction products were separated on Kinetex EVO analytical column (Kinetex 1.7 μM EVO C18 100 Å 100 × 2.1 mm, Phenominex) by applying a linear gradient of ammonium acetate/acetonitrile, as follows: 0–2 min, 0 B; 2–22 min, 0 to 20% B; 22–25 min, 20–50% B; 25–29 min, 50–100% B. Chromatographic separation was performed at 30 °C. The mobile phase A was 5 mM ammonium acetate in water, with the pH adjusted with NH_4_OH to pH = 7.0. The mobile phase B was neat acetonitrile. The flow rate was set at 0.3 ml/min. The ionization capillary voltage was set to 4500 V. The data were acquired in a scan mode in the *m/z* range of 100 to 2000.

### Reverse-phase purification of cyclic oligonucleotides

Cyclic oligonucleotides were purified from the cOA alpha–ATP reactions by reverse phase liquid chromatography (RP-LC) using a Luna Omega semi-preparative column (Luna Omega 5 μm Polar C18 100 Å 250 × 10.0 mm, Phenomenex). Reaction products were separated by a linear gradient, with % B increased from 0 to 17 in 40 min at a flow rate 5 ml/min. The mobile phase A was 50 mM triethylamine acetate (TEAA) in water, pH 7.0, and phase B was 50 mM TEAA in 80% acetonitrile. Chromatographic separation was performed at room temperature. Fractions containing cOA-ATP reaction products were concentrated using a vacuum concentrator and analyzed by ESI-MS.

### In vitro RNA cleavage assay

The RNA cleavage assays were performed with SisCsx1 or its variants (the amount of protein is indicated in each assay), 2.5 μM 5′FAM-labeled RNA, and 100 nM cOA_4_ (unless otherwise indicated) in a reaction buffer (20 mM MES pH 6.0, and 5 mM MnCl_2_). The reactions were incubated at 70 °C for the specified time periods and stopped by adding 2×stop buffer (8 M urea and 100 mM EDTA at pH 8.0) and cooling on ice. Samples were loaded on a 15% Novex TBE-urea polyacrylamide gel (Invitrogen) or a 20% denaturing gel, and analyzed according to the manufacturer′s instructions. RNA cleavage products were visualized using an Odyssey FC Imaging System (Li-Cor).

### SEC-MALS analysis

SEC-MALS experiments were performed using a Dionex (Thermo Scientific) HPLC system connected in-line to a UV detector (Thermo Scientific Dionex Ultimate 3000, MWD-3000), a Wyatt Dawn8 + Heleos 8-angle light-scattering detector and a Wyatt Optilab T-rEX refractive index detector. SEC was performed using a Superdex 200 Increase 10/300 GL column (GE Healthcare) at room temperature in a buffer containing 20 mM Tris pH 8.0, and 300 mM NaCl. For the analysis, 50 μl of Csx1, Csx1/cOA_4_, and Csx1-I383A/L390D/R391A were injected at 1.9 mg/ml, 0.4 mg/ml, and 1.9 mg/ml concentrations, respectively, and 0.5 ml/min flow rate. ASTRA (version 6.1.17) software was used to collect the data from the UV, refractive index, and light-scattering detectors. The weight average molecular masses, M_w_, were determined across the elution profile from static LS measurements using ASTRA software and a Zimm model, which relates the amount of scattered light to the weight average molecular weight of the solute, the concentration of the sample, and the square of the refractive index increment (dn/dc) of the sample.

### Binding assays

Preparation of the radioactively labelled cOA_4_ using ^32^P-α-ATP was performed with extra supplementing 0.5 μl of 3000 Ci/mmol ^32^P-α-ATP (PerkinElmer) in the reaction. The reaction was separated 24% of 19:1 acrylamide: bis-acrylamide in TBE with 8 M urea. The cOA_4_ was extracted from the gel by overnight incubation of the gel slice in MilliQ water. The binding assay was performed by adding 2 μl ^32^P-labelled cOA_4_ to increasing amount of Csx1 or its mutants from 1 to 120 nM in 20 mM MES pH 6. 0, 5 mM MnCl2 and incubating at 70 °C for 5 min, followed by analysis of 10% TBE gel (Invitrogen). The gel was visualized by phosphor imaging.

### Cooperativity and cleavage assay using dark-RNA

Dark-RNA (5′-UACUGGAUG(Fluorescein-dT)CCCCUGAA(Dabcyl-dT)GUUGC-3′BHQ1) carrying an internal fluorescein fluorophore (emission peak at 520 nm) and both an internal (Dabcyl Quenching Range: 400–550 nm) and a 3′ (BHQ1 Quenching Range: 480–580 nm) quenching molecules was synthesized by Eurofins. Increasing concentration of cOA_4_ from 1 to 128 nM were incubated in the presence of 34 nM of SisCsx1 and 0.2 μΜ dark-RNA at 70 °C for 3 min and the reactions were stopped on ice. The fluorescent released upon cleavage of the dark-RNA and the separation between fluorescein and the quenchers was measured using Varioskan™ (thermofisher) in the rage between 480 nm and 580 nm. The data points were analysed using a non-linear regression [Agonist] vs. response—Variable slope (four parameters) model from PRISM.

### Protein sequence analysis

To identify the catalytically relevant positions in Csm6 proteins, sequences from *Methanocaldococcus* and *Thermococcus* geni were aligned using Clustal Omega^34^. These sequences were used to build a profile and align the rest of Csm6 using MUSCLE^35^. To identify the hexamerization regions for both protein families, sequences of Csx1 from *Sulfolobus, Sulfurisphaera, Acidianus* and *Metallosphaera* geni were used as a profile. The same profile was used for catalytically relevant position analysis in Csx1 family. Protein sequences used in the analysis were added from the identified *csx1* and *csm6* genes by Makarova et al.^36^, together with the latest crystallized protein sequences and homologues. The amino acid frequencies in columns with aligned catalytically relevant residues were analyzed for both protein families with Unipro UGENE toolkit^37^. Identifiers for all the sequences used can be found in Supplementary Data 1, together with short summaries.

### Ring nucleases inhibition assay

Increasing concentrations of both ring-nucleases from 10 nM to 150 nM were incubated with 25 nM of cOA_4_ at 70 °C for 30 min followed by addition of 18 nM of SisCsx1 and 2.5 μΜ of RNA1 and further incubation at 70 °C for 5 min. The reactions were then separated using 15% Novex TBE-urea gel (Invitrogen).

### Cryo-EM sample preparation and data acquisition

*Sis*Csx1 sample was diluted to 0.5 mg/ml in a buffer containing 150 mM NaCl and 20 mM Tris-HCl pH 8. Then, 3 μl of sample was applied onto UltrAuFoil 300 mesh R0.6/1.0 holey grids (Quantifoil) glow-discharged for 30 s at 5 mA (Leica EM ACE200), and plunge-frozen in liquid ethane, cooled with liquid nitrogen, using a Vitrobot Mark IV robot (FEI, Thermo Fisher Scientific) with the following settings: blotting for 3 s, 100% humidity, and 4 °C. Cryo-EM data were collected using aTitan Krios G3 transmission electron microscope (FEI, Thermo Fisher Scientific) operating at 300 kV at liquid nitrogen temperature. Two datasets (dose-fractioned movies) were acquired using a Falcon 3ED direct electron detector (FEI, Thermo Fisher Scientific) in electron-counting mode, with a calibrated pixel size of 0.832 Å and nominal defocus range of −2.1 to −3.0 μm: 808 micrographs with 55 frames and a dose rate of 1.09 e^–^/Å^2^, and 1253 micrographs with 39 frames with a dose rate of 1.01 e^–^/Å^2^, respectively. EPU was used for automated data acquisition (FEI, Thermo Fisher Scientific). Cryo-EM statistics for data collection and processing are summarized in Supplementary Table 1.

### Image processing

All single-particle analyses were performed using the *cis*TEM software^38^. The micrographs were resampled to 1 Å/pixel, motion corrected by Unblur^39^, and the contrast transfer function (CTF) was estimated by CTFFIND4^40^. The images were filtered at the detected CTF fit resolution (maximum 4 Å), and 92,000 particles were picked using the automated ab initio particle picking algorithm implemented in *cis*TEM. Since a mixture of dimeric and hexameric *Sis*Csx1 particles was captured in the micrographs, the particle radii were optimized individually for the two oligomeric states in order to pick as many particles as possible. The particles were extracted using a 400 Å box and sorted by two rounds of 2D class averaging, resulting in 44k particles. An initial 3D model was generated using the *cis*TEM ab initio 3D reconstruction algorithm, which was subsequently auto-refined as one class to an overall resolution of 3.59 Å based on the gold-standard Fourier shell correlation (FSC) 0.143 cutoff criterion. The map was sharpened using the *Autosharpen Map* tool in PHENIX^41^, with a negative B-factor of 65.2 Å^2^ applied. Directional FSC analysis was performed to obtain 3D FSCs and evaluate directional resolution anisotropy^42^, and a local resolution estimation was obtained with MonoRes^43^.

### Crystallization

Initial crystallization screening using the Csx1 apo sample was performed at 298 K using the sitting-drop vapor-diffusion method and testing a collection of commercially available crystallization screens. The initial drops consisted of 0.1 μL of protein solution (7.1 mg/ml in 20 mM Tris pH 8.0, and 500 mM NaCl) and 0.1 μL well solution, and were equilibrated against 70 μL of well solution. After 3 day of incubation, the extensive initial screening rendered plate-like crystals under two different crystallization conditions. Habit I crystals were grown in 8% PEG 8000 and 0.1 M Tris pH 8.5; habit II crystals were produced in 0.350 M NaCl, 25% PEG 1000, 10% glycerol, and 0.1 M Tricine pH 8.0. Following these initial hits identification, both crystal growths were scaled up and optimized using a Dragonfly (TTP) screen optimizer. While crystallization condition II was intrinsically cryoprotectant, habit I crystals were cryo-protected by adding 35% (v/v) glycerol to the mother liquor before flash-freezing in liquid nitrogen. Iodine-modified crystals were obtained by soaking habit II Csx1 native crystals overnight in a modified mother liquor obtained by exchanging NaCl with NaI (250 mM). Csx1:cOA_4_ conformation 1 crystals were prepared by soaking at room temperature habit II Csx1 apo crystals overnight in their mother liquor solution that included 0.1 mM of the purified ligand cOA_4_. To generate Csx1:cOA_4_ crystals in conformation 2, Csx1 and the cOA_4_ ligand (2:1 molar ratio) were incubated at 343 K for 10 min before growing them in 0.05 M LiSO4, 0.1 M HEPES pH 6.5, 28% PEG 600, and 10% glycerol.

### X-ray diffraction data collection

All data were collected from frozen crystals at 100 K with EIGER and PILATUS detectors at beamlines PXI and PXIII (SLS, Villigen, Switzerland) and at BioMax (MAX-IV, Lund, Sweden). Data processing and scaling were accomplished using XDS^44^ POINTLESS and AIMLESS^45^ as implemented in autoPROC^46^. Statistics for the crystallographic data and structure solution are summarized in Supplementary Table 2.

### Crystal structure solution, model building, and refinement

Despite SisCsx1 shares a similar domain architecture with TtCsm6, SsoCsx1, or PfuCsx1, these proteins are divergent enough to make molecular replacement unsuccessful. In addition, good heavy atom derivatives were not obtained and selenomethionine substitution of SisCsx1 did not result in good diffracting crystals. Consequently, to solve the phase problem for the initial crystals belonging to P2_1_ space group, we used a medium resolution map of the SisCsx1 300 kDa complex obtained by cryo-EM as a search model in Phaser^47^. Initial molecular replacement phases where subsequently improved by solvent flattening and NCS averaging. A Cα model of SisCsx1 was built using Autobuild in Phenix^41^. This model was used in combination with SAD phasing from an iodine derivative in the I2_1_2_1_2_1_ space group in the CRANK2 pipeline^48^. The final model was fully traced in this map. The information provided by the cryo-EM map was helpful to trace some flexible loops in the crystal structure. The SisCsx1:cOA_4_ complex structure was solved by molecular replacement, as implemented in the program PHASER^47^ using SisCsx1 apo structure as the searching model. All the models were then initially subjected to iterative cycles of model building and refinement with Coot^49^, PHENIX^41^ and REFMAC^50^. Final cycle refinements were performed with BUSTER^51^ yielding the refinement and data collection statistics summarized in Supplementary Table 2. The Apo, Conf1 and Conf2 final models have a *R*_work_/*R*_free_ of 20/24, 18/23 and 19/22%, with 1.1, 1.2 and 0.7% of the residues in disallowed regions of the Ramachandran plot, respectively.

### HADDOCK ssRNA docking in the SisCsx1 catalytic pocket

HADDOCK version 2.2^26^ using CNS^52^ for structure calculations was used to dock 6-nucleotide ssRNA in the catalytic pocket of the HEPN domain. The crystal structures of the apo, conf1 and conf2 SisCsx1 were used to extract the initial coordinates of the protein for the docking and to identify the residues defining the catalytic pocket. Residues with atoms with centers within 10 Å of H404 and with all-atom relative solvent accessibility greater than 20% define the catalytic pocket. This comprises residues 348, 354, 370–373, 389–393, 399, 400–404, and 410–415 of each SisCsx1 monomer. The linear molecule coordinates for the ssRNA docking were generated in coot^49^. The default version topology and parameter files provided for proteins in HADDOCK 2.2 were used to generate the protein and ligand structures. Briefly, 1000 docked complex structures were generated in the first rigid-body docking step (it0), 200 structures in the semi-flexible simulated annealing (it1) and 200 structures evaluated in the final analysis. Two sets of distance restraints were used at different stages of the docking protocol. For the rigid-body docking, it0, the entire binding pocket and the ligand were defined as active. For the flexible refinement steps, the binding pocket was defined as passive while the ligand was defined as active. Molecular dynamics simulations were switched off (the number of MD steps was set to 0) for both rigid-body high temperature docking and the slow cooling annealing step of the semi-flexible simulated annealing. HADDOCK score was used to rank the models. Models were clustered using the RMSD criteria with a 2.0 Å cutoff. The structures of the top clusters with lowest HADDOCK scores were examined manually in PyMOL^53^.

### Visualization

Figures were generated using PyMOL^53^, Chimera, and ChimeraX^54^.

### Reporting summary

Further information on research design is available in the Nature Research Reporting Summary linked to this article.

## Supplementary information

Supplementary Information

Peer Review File

Description of Additional Supplementary Files

Supplementary Data 1

Supplementary Movie 1

Supplementary Movie 2

Supplementary Movie 3

Supplementary Movie 4

Supplementary Movie 5

Reporting Summary

Source Data

## Data Availability

The EM map has been deposited in the EMDB under the code EMDB-4691. Atomic coordinates and structure factors have been deposited in the Protein Data Bank under the accession codes 6QZT [https://www.rcsb.org/structure/6QZT], 6R7B [https://www.rcsb.org/structure/6R7B], and 6R9R [https://www.rcsb.org/structure/6R9R], accordingly. The source data underlying Figs. 4e–g, Fig. 6c and Supplementary Figs. 5A–C, 8A–C are provided as a Source Data file. The enquiry on genetic materials generated in this work should be addressed to QS. All other data are available from the corresponding author upon reasonable request.
