# Supplementary material for: Structure of Csx1-cOA4 complex reveals the basis of RNA decay in Type III-B CRISPR-Cas
Source: Nat Commun. 2019 Sep 20;10:4302. doi: 10.1038/s41467-019-12244-z (PMC6754442; doi:10.1038/s41467-019-12244-z)
Supplement: Supplementary file 1 — Supplementary Information [file 41467_2019_12244_MOESM1_ESM.pdf]

Supplementary Information

Structure of Csx1-cOA$_4$ complex reveals the basis of RNA decay in Type III-B CRISPR-Cas

Rafael Molina, Stefano Stella, Mingxia Feng et al.,

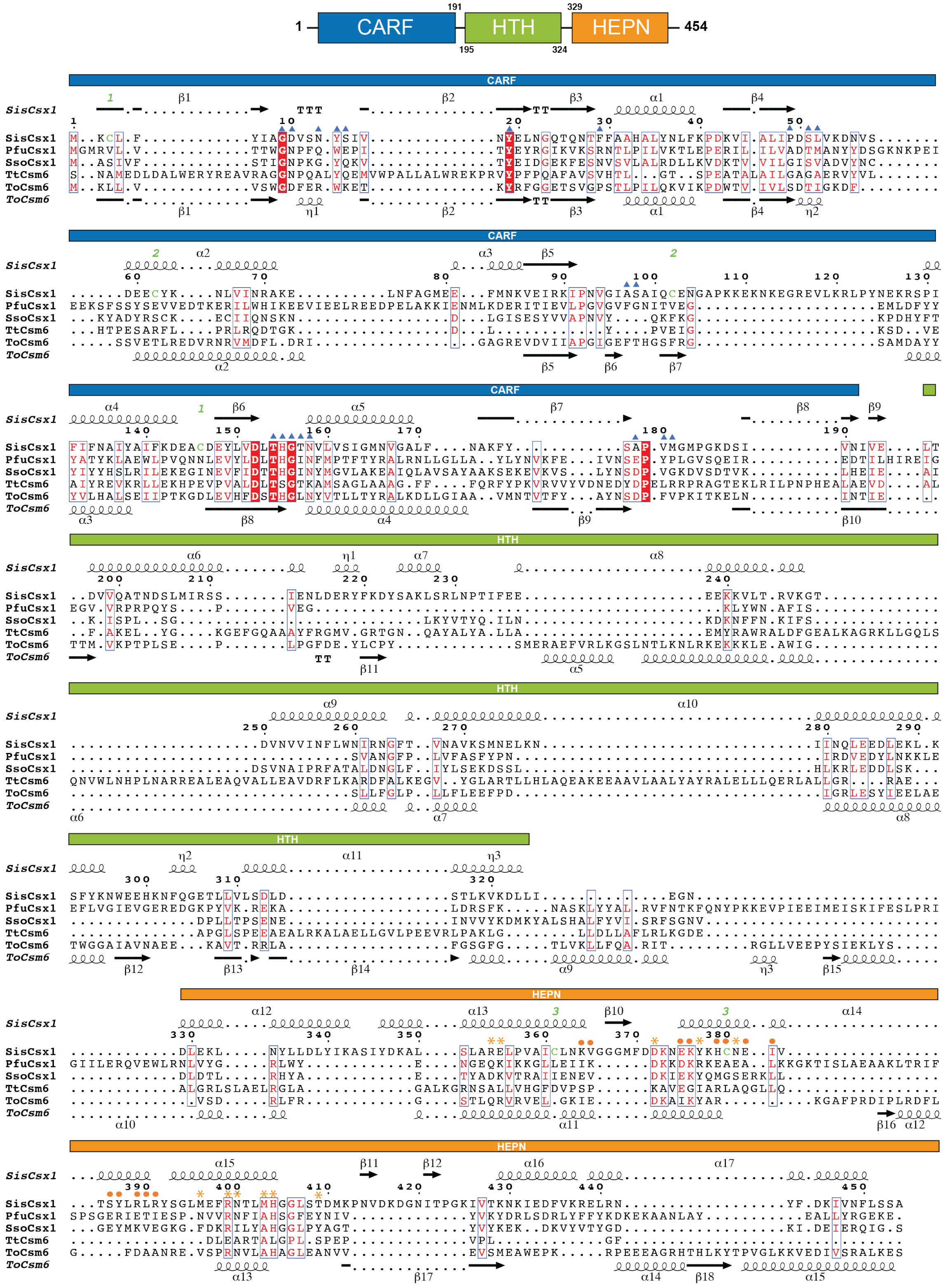

Supplementary Figure 1. Structure-based sequence alignment of type III-B CRISPR-associated CARF ribonucleases. The amino acid sequences of Csx1 from *S. islandicus* (SisCsx1), *Pyrococcus furiosus* (PfuCsx1), *Sulfolobus sulfataricus* (SsoCsx1), *Thermus termophilus* (TtCsm6) and *Thermus onnurineus* (ToCsm6) were aligned by using Clustal Omega (https://www.ebi.ac.uk/Tools/msa/clustalo). The figure was prepared using ESPript (http://espript.ibcp.fr/ESPript/ESPript/index.php). Residue numbers are labelled according to the SisCsx1 sequence. The different structural domains of SisCsx1 and their amino acid composition are depicted as boxes above the sequences, and labelled with the same names and color code as in Fig. 1A. Key residues for cOA_4 binding, RNA catalysis, and Csx1 oligomerization are marked according to their region: CARF domain (blue triangle), HEPN domain (orange stars), and hexamer insertion region (orange dots), respectively. Residues involved in intramolecular sulphur bridge bonds are displayed along the SisCsx1 sequence by green numbers (1, 2 and 3).

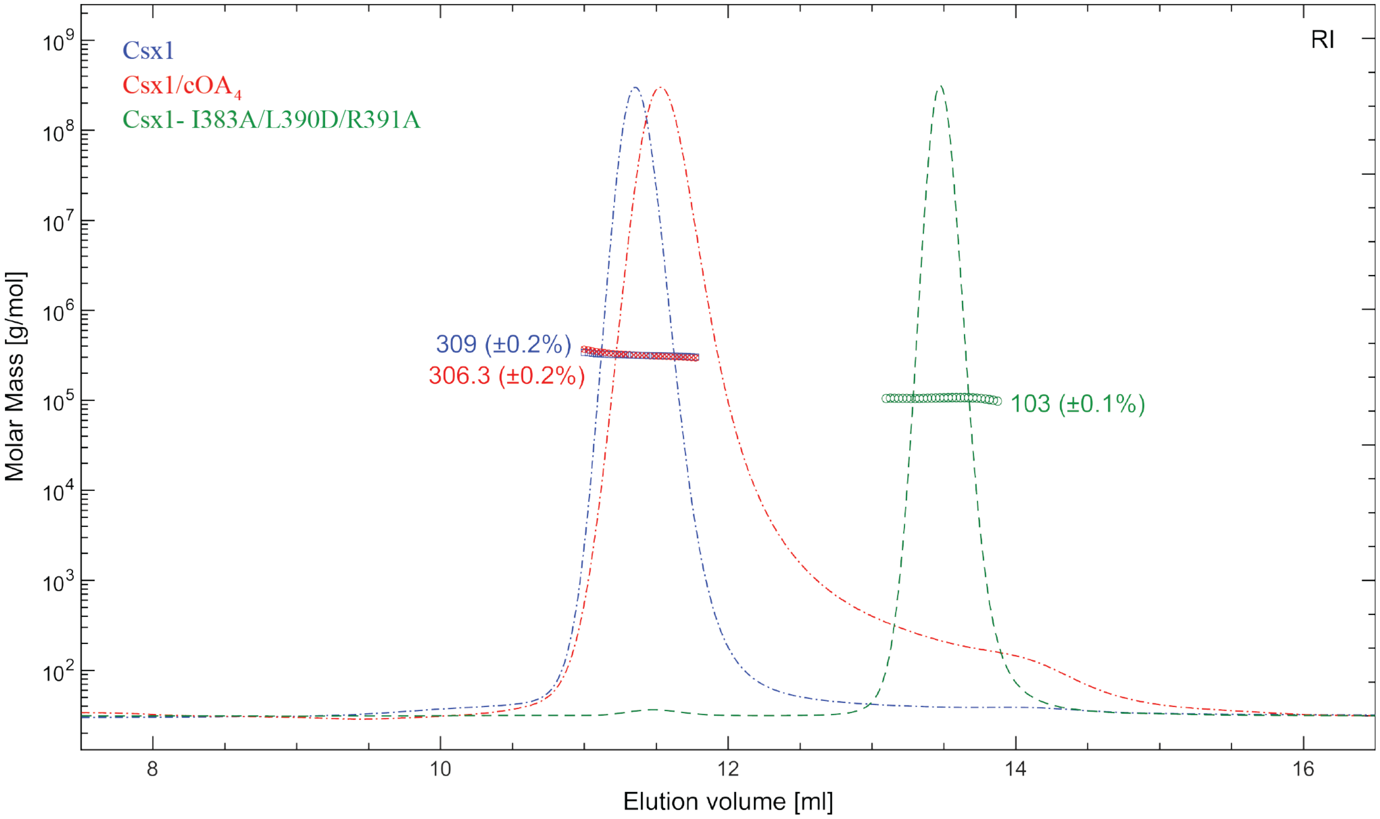

Supplementary Figure 2. SEC-MALS analysis. SisCsx1, SisCsx1/cOA$_4$ complex, and SisCsx1-I383A/L390D/R391A variant were injected into a Superdex 200 Increase 10/300 GL column. The change in refractive index as a function of protein concentration was used to compute the molar masses for the different samples. The discontinued lines plotted on the right axis scale correspond to the RI traces from the SEC column scaled across the greatest magnitude of all chromatogram data. The molar masses across the eluting peaks are plotted as open circles on the left axis scale (molar mass). The average molecular weights are displayed on the figure.

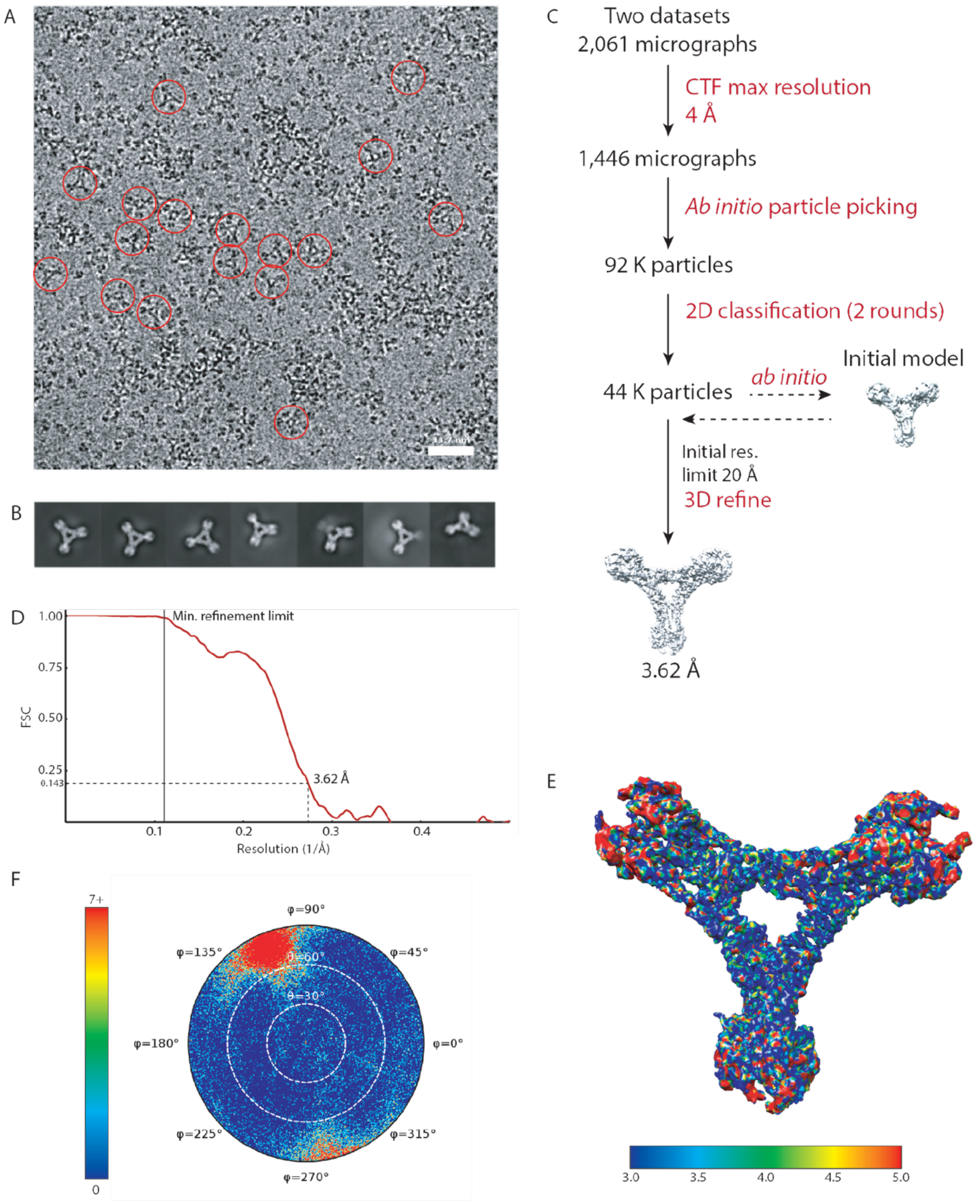

Supplementary Figure 3. Cryo-EM data processing and reconstruction. (A) Representative cryo-EM micrograph of the Csx1 complex. A few picked particles are highlighted in red circles. (B) Reference-free 2D class averages selected for further processing. (C) Overview of the cryo-EM data processing workflow for the Csx1 complex in cisTEM. Marked up in red: Filtering of the micrographs based on the estimated contrast transfer function (CTF) maximum resolution, as well as the data processing job types performed, including particle picking, 2D classification, *ab initio* reference model generation, and 3D refinement. (D) The particle Fourier shell correlation (FSC) curve of the final 3D reconstruction. The minimum refinement limit used for the final 3D reconstruction was 8.5 Å.(E) Local resolution map calculated using MonoRes within the Scipion package. (F) Euler angular distribution plot of the relative orientations of all particles included in the final 3D reconstruction of the Csx1 complex.

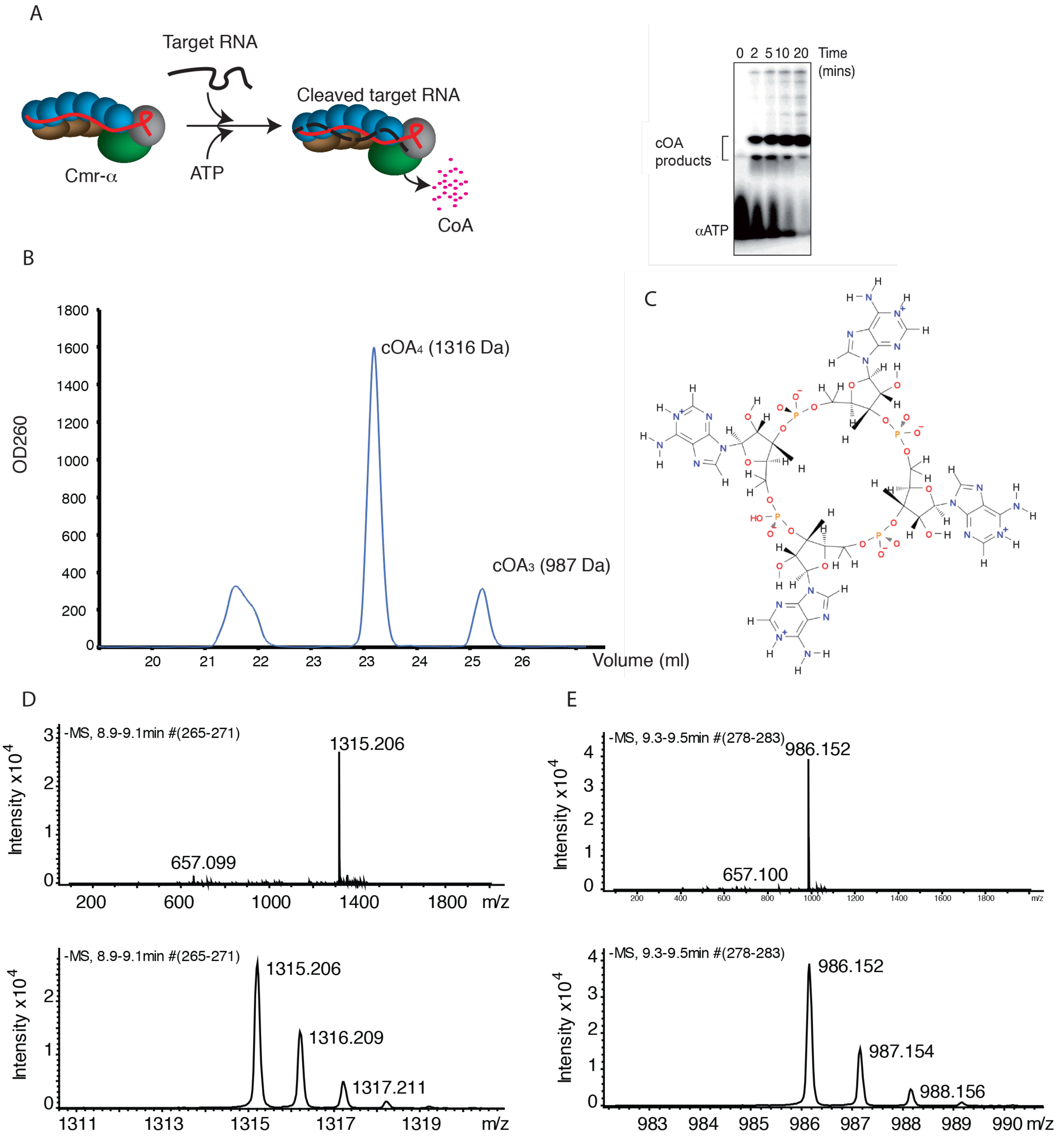

Supplementary Figure 4. cOA_4 production and purification including ESI-MS analysis of LC elution peaks. **(A)** cOA were generated in vitro using Cmr-α-RNP from *S. islandicus* Rey15A was performed as described [35]. The gel sows the presence of two main products after the reaction. **(B)** The two cOA species were separated by reverse phase HPLC. The molecular weights of 1316 Da, and 987 Da indicate that they were composed of 4 and 3 AMPs respectively. **(C)** Drawing of the cOA_4 chemical formula. Mass spectra of the compounds and the isotopic patterns of the cOA_4 **(D)** and cOA_3 **(E)** detected compounds.

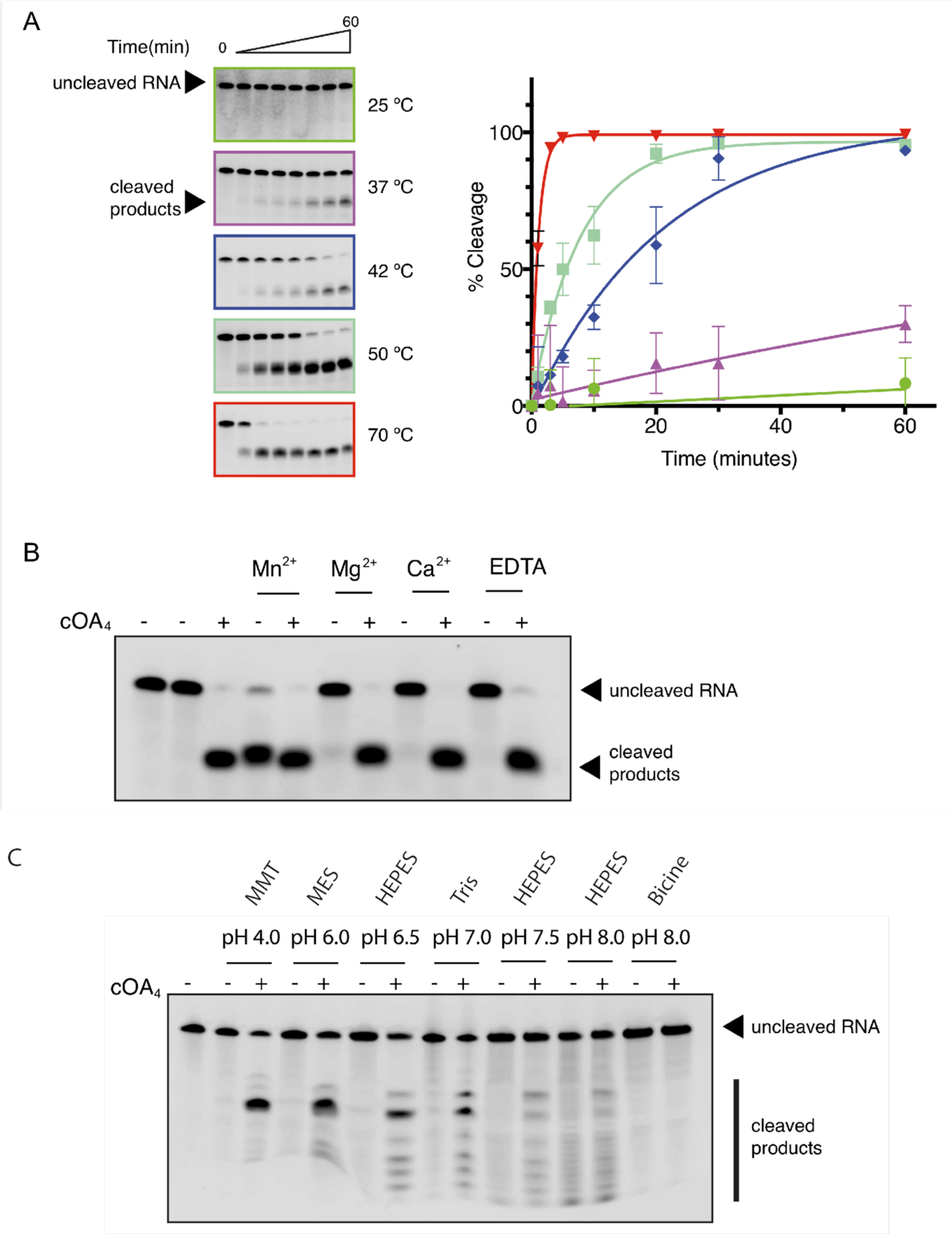

Supplementary Figure 5. Temperature effect on SisCsx1 activation in the presence of cOA_4. **(A)** Gels and chart displaying the activity of SisCsx1 measured at different temperatures in the presence of 100 nM cOA_4, 18 nM SisCsx1 and 2.5 µM of RNA9 (see Fig. 6C) as substrate. **(B)** Effect of the presence of metals on SisCsx1 activity. The experiment conditions were the same as in (A)**. (C)** Effect of the pH on SisCsx1 activity shows that high pHs promoting histidine protonation do not support cleavage. The experiment conditions were the same as in (A) except the different buffers and that the substrate is RNA1 (see Fig. 6C). The experiments were repeated three times and the bars in the chart represent the s.d. Source data are provided as a Source Data file for Supplementary Figs 5A-C.

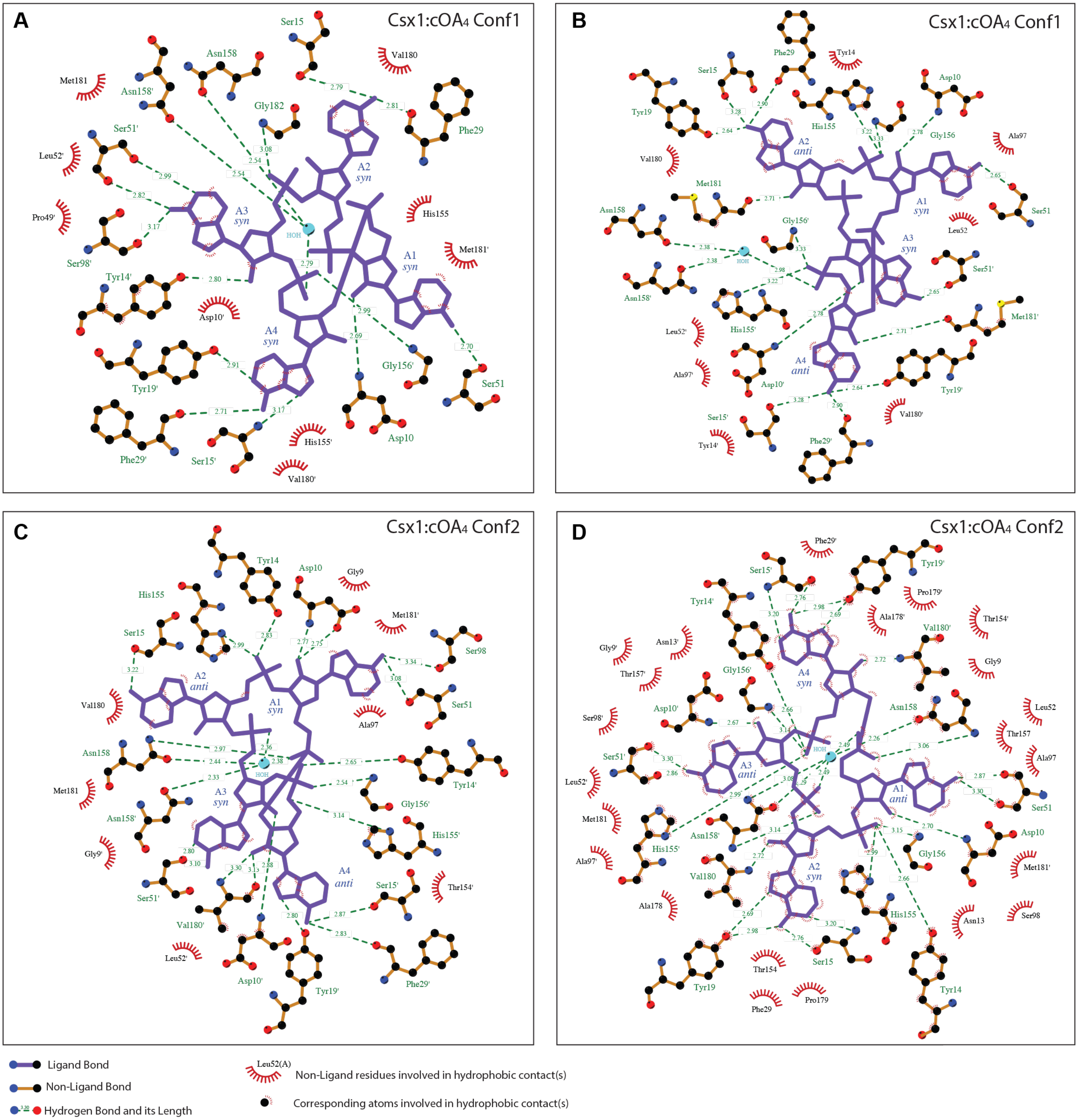

Supplementary Figure 6. Schematic representation of cOA_4 interactions in SisCsx1 second messenger binding pockets. The interactions were depicted by LigPlot in conf1 **(A-B)** and conf2 **(C-D)**.

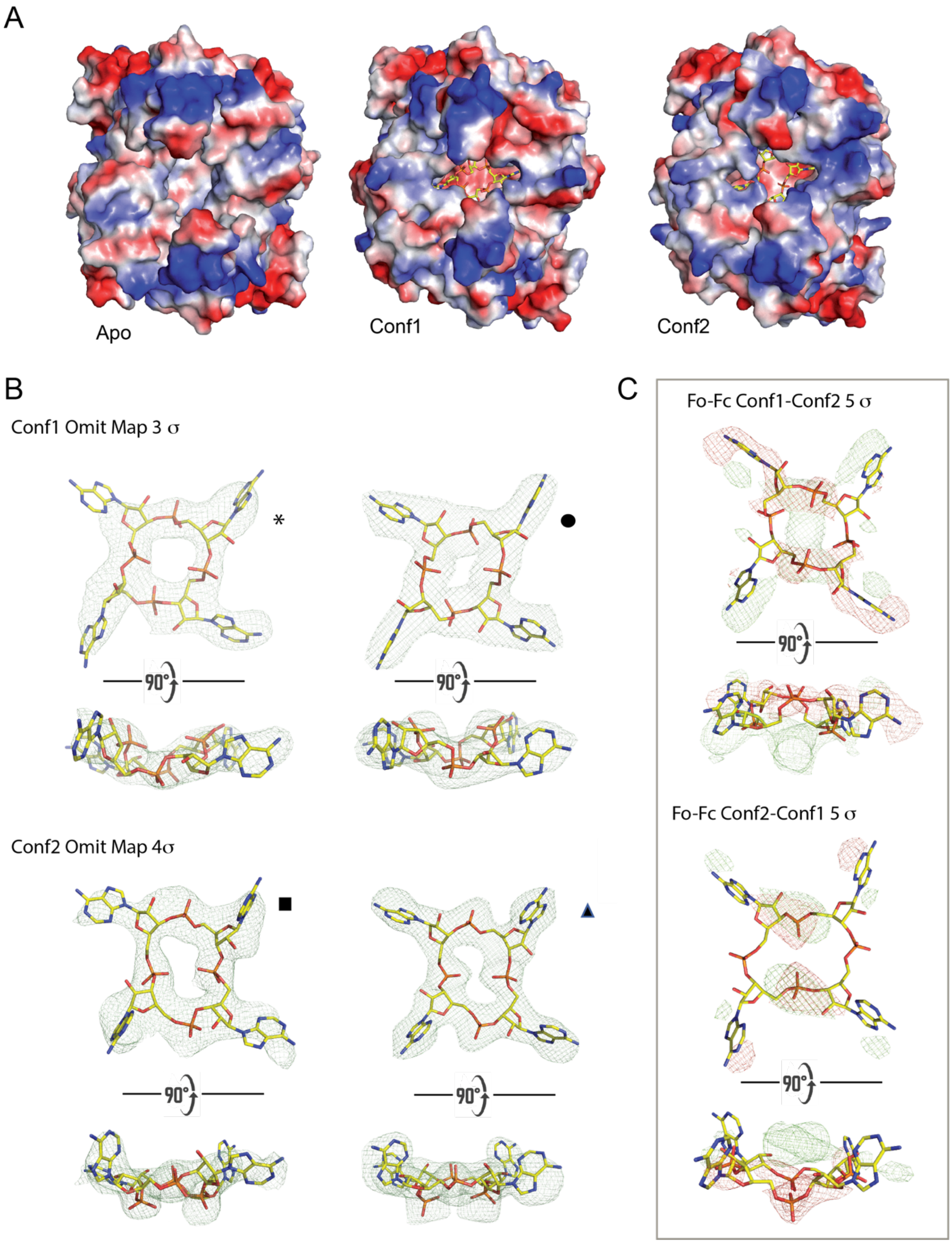

Supplementary Figure 7. (A) Electrostatic potential of the cOA_4-binding pocket. Top view of a SisCsx1 dimer in the apo, conf1, and conf2 structures. The detailed view shows how the shape in one of the pockets and the electrostatic potential in the cleft change upon binding and the conf1-conf2 transition. (B) The figure shows the Fo-Fc omit maps of the cOA_4 molecule in conf1 and conf2. (C) Fo-Fc difference maps. The figure shows the negative peaks in the Fo-Fc electron density when the cOA_4 conformations conf1 and conf2 are exchanged (red negative, green positive), thus validating the observed conformational changes. Maps are displayed at $\pm 5.0\ \sigma$ contour value.

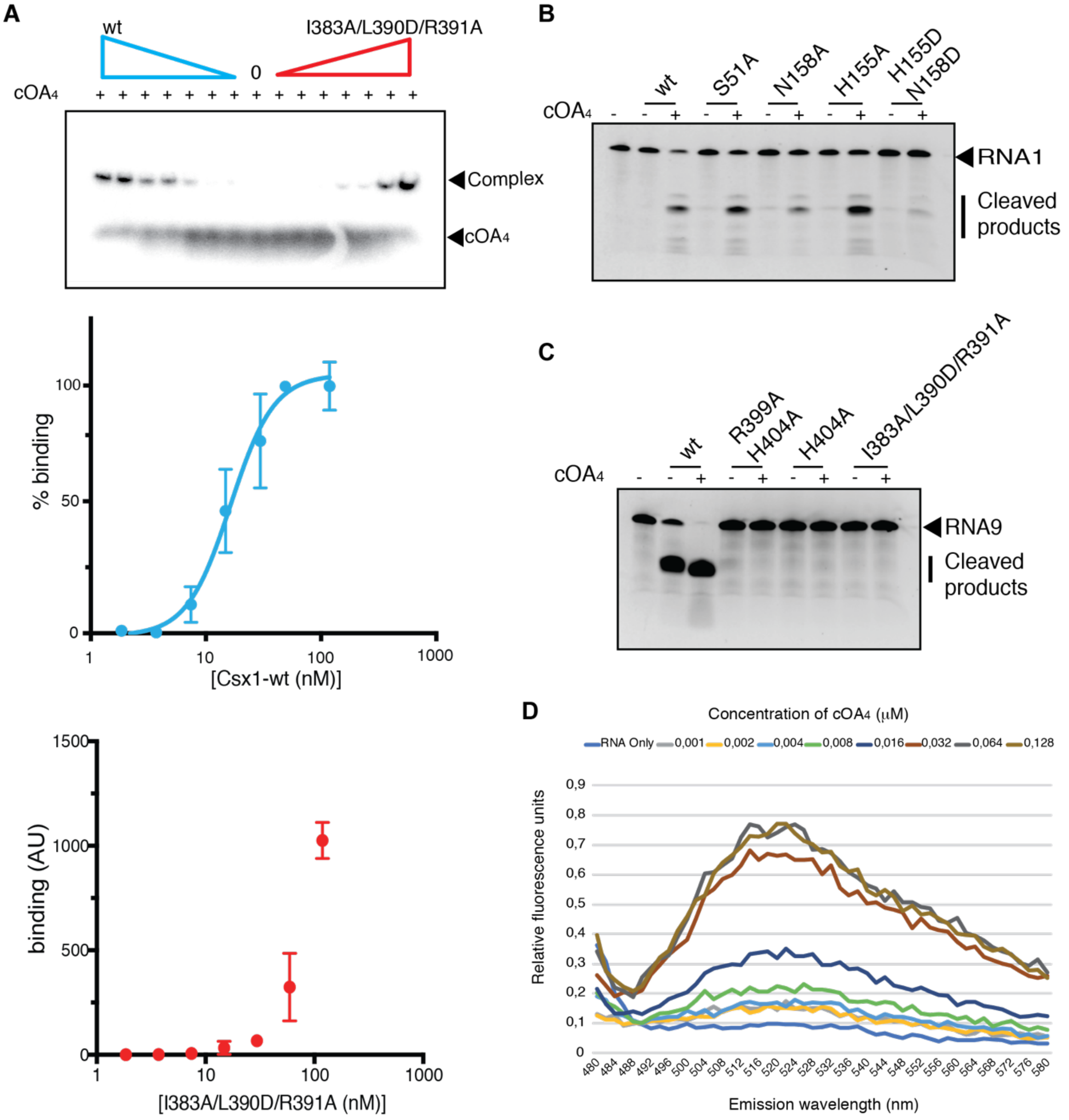

Supplementary Figure 8. SisCsx1 and I383A/L390D/R391A mutant cOA4 binding and CARF and HEPN mutant ssRNase activity. (A) Binding assays of SisCsx1 and I383A/L390D/R391A variant. The experiments were performed as described in Materials and Methods. Quantification of the bands corresponding to the complex between the proteins and cOA4 from three independent experiments are plotted below. Curve fitting of SisCsx1 binding was fitted using a non-liner regression specific binding model as implemented in PRISM. A K_d of ~18 nM was calculated for cOA4 binding by SisCsx1. As there is no saturation for I383A/L390D/R391A mutant binding, it is not possible to plot a % scale. (B) *in vitro* RNA cleavage assay of the SisCsx1 CARF domain variants. 1 μM of RNA1 (see Fig. 6C) was incubated at 70˚C for 1 minute in the presence of 25nM of cOA4 and 18nM of corresponding proteins. The cleavage reactions were separated on 15% Novex TBE-urea polyacrylamide gel. (C) *in vitro* RNA cleavage assay of the SisCsx1 HEPN domain variants. 2.5 μM of RNA9 (see Fig. 6C) was incubated at 70˚C for 5 minutes in the presence of 100nM of cOA4 and 18nM of corresponding proteins. The cleavage reactions were separated on 15% Novex TBE-urea polyacrylamide gel. (D) Cooperativity experiment raw data. The chart shows one of the typical experiments to assess cooperativity. The increase in fluorescence is generated by the cleavage of the ssRNA substrate in different concentrations of cOA4. Briefly, increasing concentration of cOA4 from 1 to 128 nM were incubated in the presence of 34 nM of SisCsx1 and 0.2 μM dark-RNA at 70 °C for 3 minutes and the reactions were stopped on ice (Methods). The experiments were repeated three times and the bars in the chart represent the s.d. Source data are provided as a Source Data file for Supplementary Figs 8A-C.

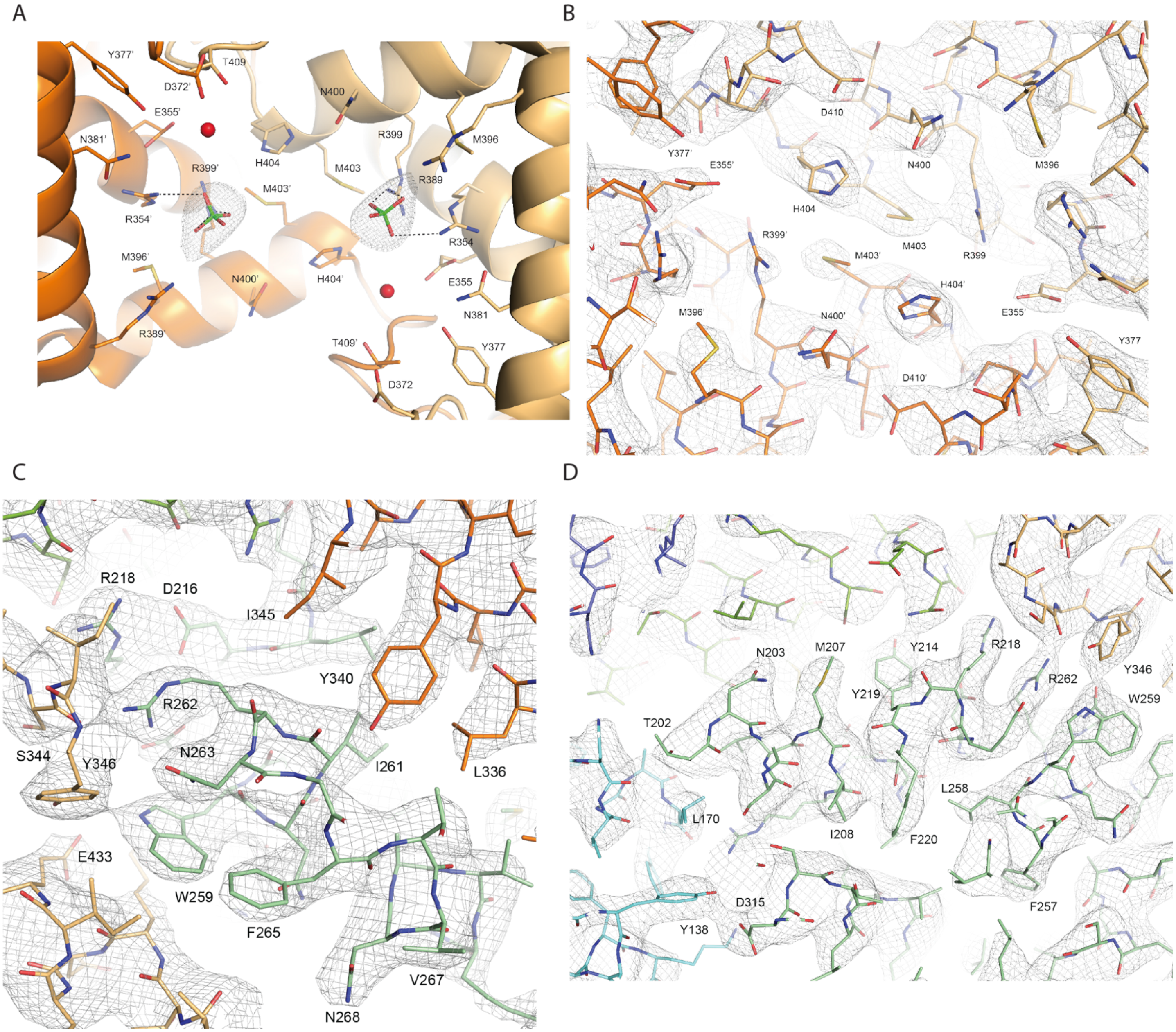

Supplementary Figure 9. (A) Detection of sulfate atoms in the catalytic pocket in conf2. The Fo-Fc map displays sulphate ions (1.5 σ) bound to both R354 and R399 in the catalytic pocket suggesting that they can be involved in ssRNA phosphate backbone binding to promote cleavage. 2fo-fc electron density map contoured at 1.1 σ show the quality of the structures. (B) The zoom shows the catalytic centre in the HEPN domain. The catalytic H404 residues are labelled in the centre. (C-D) The zooms show 2fo-fc electron density of the region between the HTH (green) HEPN (orange) and CARF (blue) domains. The ´symbol differentiates residues from the two monomers.

Supplementary Table 1. Cryo-EM statistics for data collection and processing.

	Csx1 hexamer
**EMDB code**	4691
**Data collection and processing**	
Magnification	96,000
Voltage (KV)	300
Electron exposure ($e^{-}/Å^2$)	40 or 60
Defocus range (μm)	-2.1 to -3.0
Pixel size (Å)	0.8320
Initial particles (no.)	93232
Final particles (no.)	43715
Map resolution (Å)	3.62
FSC threshold	0.143
Map sharpening B factor ($Å^2$)	65.2

In the analysis, 808 micrographs received 60 e-/Å2, and 1253 received 40 e-/Å2. Csx1: 400-pixel box, resampled to 1 Å/px.

Supplementary Table 2. X-ray crystallographic data collection and refinement statistics.

	Csx1 native crystal condition I	Csx1:cOA$_4$ Conformation 1	Csx1:cOA$_4$ Conformation 2
**PDB code**	6QZT	6R7B	6R9R
**Data collection**			
X-ray source	PXI (SLS)	PXI (SLS)	PXI (SLS)
Space group	$I2_12_12_1$	$I2_12_12_1$	$I2_12_12_1$
Cell dimensions			
a, b, c (Å)	107.62, 118.80, 357.68	107.08, 117.68, 357.26	104.48, 119.52, 358.09
α, β, γ (°)	90, 90, 90	90, 90, 90	90, 90, 90
Wavelength	1.00	0.98	1.00
Resolution (Å)	112.74–2.79 (3.03–2.79)*	178.63–3.12 (3.46–3.12)*	78.62–2.70 (2.88–2.70)*
CC(1/2)	0.99 (0.68)	0.99 (0.50)	0.99 (0.50)
Mean I / σI	13.6 (1.7)	11.7 (1.4)	17.7 (1.4)
Completeness (%)[#]	88.2 (68.6)	93.0 (56.5)	93.5 (81.2)
Redundancy	14.1 (13.1)	11.6 (8.7)	8.8 (8.7)
**Refinement**			
Resolution (Å)	39.32–2.93	48.29–3.12	52.24 –2.70
No. Reflections	33959	24506	44348
R_{work} / R_{free}	0.20/0.24	0.18/0.23	0.19/0.22
No. Molecules in a.u.	3	3	3
No. Atoms			
Protein	10926	10926	10926
cOA$_4$	0	132	132
Water	264	95	228
Ramachandran			
Favored/Allowed (%)	98.9	98.8	99.3
Disallowed (%)	1.1	1.2	0.7
R.m.s. deviations			
Bond lengths (Å)	0.010	0.010	0.009
Bond angles (°)	1.230	1.260	1.170

*Values in parentheses are for highest-resolution shell. One crystal was used to solve each structure.

[#]Ellipsoidal Completeness according to STARANISO [26] cutoff criteria.

Supplementary Table 3. Hydrogen bonds in the HEPN domain interactions between dimers in the apo, conf1, and conf2 structures.

Apo

Dimer 1		Dimer 2		Dimer 3	
Chain B	Chain E	Chain A	Chain C	Chain D	Chain F
R391	K364<br>A454	R391	K364<br>A454	R391	K364<br>A454
S453	S453				
		K446	S453	K446	S453
K364<br>A454	R391	K364	R391	K364	R391
		A454	R391<br>Y387	A454	R391<br>Y387
		H379	S386	H379	S386
		S386	H379	S386	H379

Conf1

Dimer 1		Dimer 2		Dimer 3	
Chain B	Chain F	Chain A	Chain I	Chain G	Chain H
R391	K364<br>A454	R391	K364<br>A454	R391	A454
S453	S453	S453	S453	S453	S453
		K446	S453	K446	S453
K364<br>A454	R391			K364	R391
		A454	R391<br>Y387	A454	R391<br>Y387
		H379	S386	H379	S386

Conf2

Dimer 1		Dimer 2		Dimer 3	
Chain B	Chain F	Chain A	Chain I	Chain G	Chain H
R391	K364	Y387	S453<br>A454	Y387	S453<br>A454
S453	S453	S453	S453	S453	S453
		K446	S453	K446	S453
A454	K446<br>R391				
Y387<br>K446	A454	A454	R391	A454	R391
K364	R391	E382	S386	E382	S386
		S386	H379	S386	H379

Supplementary Table 4. *Sulfolobus* strains used in this work.

Strains	Genotype and features	Reference
S. islandicus **E233S1**	***ΔpyrEFΔlacS***	[1]
S. islandicus **E233**	***ΔpyrEF***	[1]
S. islandicus Δα	**Derived from E233. With the deletion of the IIIB Cmr-α locus including six genes**	[5]
S. islandicus Δcsx1Δα	**Derived from *S. islandicus Δα*. With the deletion of *csx1***	**This work**

Supplementary Table 5. Plasmids used in this work.

Plasmids	Genotype and features	Reference
**pSe-RP**	**Cloning vector for constructing mini-CRISPR arrays for *Sulfolobus***	[4]
**pAC-csx1-S1**	**Artificial mini-CRISPR locus plasmid derived from pSe-Rp, carrying an artificial CRISPR locus with S1 spacer of the *S. islandicus csx1* gene**	**This work**
**pGE-csx1**	**Genome editing plasmid for the deletion of *csx1* gene in *S. islandicus***	**This work**
**pSe-Csx1**	**Expression plasmid for wild-type Csx1 protein in *S. islandicus***	[6]

Supplementary Table 6. Oligonucleotides used in this work.

**Oligonucleotides**	**Sequence** (5′–3′)
Del csx1-F-SalI	TTACGCGTCGACTATTCTCATGTCCTAAGTGC
Del csx1-SOE-R	AGACCTGAATACCTTAGCCTATAACACTCCTCATCGCTTA
Del csx1-SOE-F	TAAGCGATGAGGAGTGTTATAGGCTAAGGTATTCAGGTCT
Del csx1-R-NotI	AAGGAAAAAAGCGGCCGCTGTCTTCATTTTTAGTGGTA
Del csx1spacerI-F	AAAGTGAAATACCTCTCGTCCAAATTTTCTATGGAGGATCTTAT
Del csx1spacerI-R	TAGCATAAGATCCTCCATAGAAAATTTGGACGAGAGGTATTTCA
0884H155A-F	GACTTAACTGCAGGTACAAACGTCCTA
0884H155A-R	GTTTGTACCTGCAGTTAAGTCTACTAA
0884S51A-F	TCCCGATGCATTAGTAAAAGACAATGTAAGCGATG
0884S51A-R	ACTAATGCATCGGGAATTAATGCAATTAC
0884N158A-F	TGGTACAGCAGTCCTAGTATCTATTGGGATGAACG
0884N158A-R	AGGACTGCTGTACCATGAGTTAAGTCTACTAAA
0884I383A/L390D/R391A-F	AACTTCTTATCTAAGGGACGCATATTCAGGTCTCATGGAATTTAGG A
0884I383A/L390D/R391A-R	CTTAGATAAGAAGTTACTGCTTCATTACAGTGTTTGTATTTTTCA
0884H155D/N158D-F	CTGATGGTACAGACGTCCTAGTATCTATTGGGATGAACG
0884H155D/N158D-R	CGTCTGTACCATCAGTTAAGTCTACTAAATATTCGTCG
0884H404A-F	ACACTGATGGCAGGAGGATTGTCAAC
0884H404A-R	CAATCCTCCTGCCATCAGTGTATTCC
