# Supplementary material for: Structure of Csx1-cOA4 complex reveals the basis of RNA decay in Type III-B CRISPR-Cas
Source: Nat Commun. 2019 Sep 20;10:4302. doi: 10.1038/s41467-019-12244-z (PMC6754442; doi:10.1038/s41467-019-12244-z)
Supplement: Supplementary file 3 — Description of Additional Supplementary Files [file 41467_2019_12244_MOESM3_ESM.pdf]

**Description of Additional Supplementary Files**

**File name:** Supplementary Data 1

**Description:** Genomic Analysis of prokaryotic genomes containing Csx1 and Csm6 RNase genes. The analysis summarizes the presence of the residues involved in oligomerization in the HEPN domain and those involved in a possible degradation of $cOA_4$ in the CARF domain.

**File name:** Supplementary Movie 1

**Description:** Morphing showing the conformational change in the $cOA_4$ binding site between the SisCsx1 apo, conf1 and conf2 structures. Each CARF domain is colored according to Fig. 3. The side chains of key residues are depicted in the morphing.

**File name:** Supplementary Movie 2

**Description:** Morph between the apo, conf1 and conf2 structures showing how the conformational change in the $cOA_4$ binding site is transmitted from the CARF domain in one monomer to the HTH domain belonging to the other monomer within the dimer (See Fig. 1C and Figure 2B). The colors correspond to those in Fig. 1C.

**File name:** Supplementary Movie 3

**Description:** Morph between the apo, conf1 and conf2 structures showing how the conformational change is transmitted within the hexamer to the other catalytic centers through the HTH and the pins in the HEPN domains. The colors correspond to those in Fig 5A, D, E.

**File name:** Supplementary Movie 4

**Description:** Morphing showing an overall view of the conformational change between SisCsx1 apo, conf1 and conf2 in one of the dimers showing how the conformational changes in $cOA_4$ are transmitted from the CARF to the HTH and finally to the HEPN domains. The second part of the Supplementary videohows how this conformational change is transmitted inside the SisCsx1 hexamer.

**File name:** Supplementary Movie 5

**Description:** Morphing between the apo, conf1 and conf2 structures in the RNase catalytic site showing how the site is compacted by the conformational change and the catalytic residues (H404 and H404′) change their rotamers, while those supposed to hold the phosphate backbone (R354, R354′, R399, R399′, N400, N400′) are more static.
