# Supplementary material for: Structure of Csx1-cOA4 complex reveals the basis of RNA decay in Type III-B CRISPR-Cas
Source: Nat Commun. 2019 Sep 20;10:4302. doi: 10.1038/s41467-019-12244-z (PMC6754442; doi:10.1038/s41467-019-12244-z)
Supplement: Supplementary file 2 — Peer Review File [file 41467_2019_12244_MOESM2_ESM.pdf]

Editorial Note: This manuscript has been previously reviewed at another journal that is not operating a transparent peer review scheme. The manuscript was considered suitable for publication without further review at *Nature Communications.*
